# A pilot randomized placebo-controlled study on modified MaZiRenWan: a formulated Chinese medicine to relieve constipation for palliative cancer patients

**DOI:** 10.1186/s13020-022-00580-0

**Published:** 2022-03-02

**Authors:** Chung-wah Cheng, Hoi-fung Mok, Cora W. S. Yau, Jasmine T. M. Chan, Yu-chen Kang, Pui-yan Lam, Linda L. D. Zhong, Chen Zhao, Bacon F. L. Ng, Annie O. L. Kwok, Doris M. W. Tse, Zhao-xiang Bian

**Affiliations:** 1 Hong Kong Chinese Medicine Clinical Study Centre, School of Chinese Medicine, Hong Kong Baptist University, 3/F, Jockey Club School of Chinese Medicine Building, 7 Baptist University Road, Hong Kong, SAR People’s Republic of China; 2 Yan Chai Hospital – Hong Kong Baptist University Clinical Centre for Training and Research in Chinese Medicine (West Kowloon), Yan Chai Hospital, Hong Kong, SAR People’s Republic of China; 3 Integrated Palliative Care Unit, Department of Medicine, Hong Kong Buddhist Hospital, Hong Kong, SAR People’s Republic of China; 4 Palliative Care Unit, Department of Medicine and Geriatrics, Our Lady of Maryknoll Hospital, Hong Kong, SAR People’s Republic of China; 5 Hong Kong Buddhist Association—University of Hong Kong Clinical Centre for Teaching and Research in Chinese Medicine, Hong Kong, SAR People’s Republic of China; 6 Institute of Basic Research in Clinical Medicine, China Academy of Chinese Medical Sciences, Beijing, China; 7 Chinese Medicine Department, Hospital Authority, Hong Kong, SAR People’s Republic of China; 8 Palliative Care Unit, Department of Medicine and Geriatrics, Caritas Medical Centre, 111 Wing Hong Street, Shamshuipo, Hong Kong, SAR People’s Republic of China

**Keywords:** Constipation, MaZiRenWan, Traditional Chinese Medicine, Palliative care, Randomized controlled trial, Placebo

## Abstract

**Background:**

Constipation is a common problem among advanced cancer patients; however, many of them find limited effective from current therapies. Thus, we aimed to test the effect of a traditional Chinese herbal formula, modified MaZiRenWan (MZRW), by comparing with placebo among palliative cancer patients with constipation.

**Methods:**

This is a randomized, double-blind, placebo-controlled trial. Participants aged over 18 were recruited and randomized to MZRW or placebo group in addition to current prescriptions (including ongoing laxatives treatment) for two weeks. Exclusion criteria included cognitive impairment, presence of a colostomy or gastrointestinal obstruction and estimated life expectancy of less than one month. Individualized modification of MZRW was allowed according to the traditional Chinese medicine (TCM) pattern of patient. The primary outcome was the global assessment of improvement, which reflected whether the constipation had improved, remained the same or worsened.

**Results:**

Sixty patients, with mean age 75.2 years (range 47–95 years), were randomized to MZRW or placebo group. Among the MZRW group, 59.3% (16/27) had improvement in the global assessment score, as compared with 28.6% (8/28) of the placebo group (p-value = 0.022). Besides, the MZRW group had significant increase in stool frequency, and reduction in constipation severity and straining of defecation (p-value < 0.05). No serious adverse event was reported due to the research medication.

**Conclusion:**

This pilot trial suggests modified MZRW is well-tolerated and effective for relief of constipation in patients with advance cancer. It could be considered as a potential treatment option for constipation in palliative care.

*Trial registration*: The trial had been registered in ClinicalTrials.gov with identifier number NCT02795390 [https://clinicaltrials.gov/ct2/show/NCT02795390] on June 10, 2016.

**Supplementary Information:**

The online version contains supplementary material available at 10.1186/s13020-022-00580-0.

## Background

Constipation is a common problem in advanced cancer patients with prevalence between 40 and 90% [1]. It is a major cause of distress and may develop to life-threatening impaction with circulatory, cardiac or respiratory symptoms [2]. The European Society for Medical Oncology (ESMO) Clinical Practice Guideline suggests that the best practice is based on a balance between non-pharmacological (prevention and self-care) and pharmacological strategies (oral and rectal laxative therapy) [1]. The stepwise approach consists of first osmotic or stimulating laxatives, second a combination of two classes of laxatives, then peripheral opioid antagonists, and last by adding any pharmacological or non-pharmacological measures [3]. However, assessment and effective treatment of constipation remained a challenge in clinical practice. In a study involving 17 centres across Europe, 60% of 1,938 patients receiving strong opioids was inadequately treated [4], and underestimation of symptom by healthcare provider was among the risk factors for inadequate treatment. In our previous cross-sectional study in 225 advanced cancer patients, 80.4% of them reported constipation despite more than 60% had already been prescribed laxatives. Near 30% of patients in severe constipation reported with more inadequate pushing force, sense of incomplete defecation or difficult defecation, but no difference in stool types as assessed by the Bristol Stool Scale by comparing with the rest of the cohort [5]. Therefore, a safe and effective remedy for constipation is in great demand for cancer palliative care.

Traditional Chinese medicine (TCM) focuses on correcting maladjustments and restoring the self-regulatory ability of the human body [6, 7], which gains increasing attention in the field of palliative medicine [8]. According to the TCM theory, constipation can be broadly divided into excess and deficiency patterns by differentiating underlying symptoms and signs [9]. MaZiRenWan (MZRW), also called Hemp Seed Pill, is a classic Chinese herbal formula widely used for constipation. We found that MZRW was useful for alleviating functional constipation for subjects in excess pattern when compared with placebo and senna [10–12]. In our earlier study on advanced cancer patients with constipation, deficiency pattern was common, and often complicated by excess pattern [9]. The findings suggested that modification of MZRW could be useful for relief of constipation in advanced cancer patients, and this study would provide evidence on its efficacy as compared with placebo.

For better making use of TCM in the palliative care, further support with scientific proof is essential [13, 14]. We believe that the results can provide robust clinical evidence to implement a large scale randomized controlled trial (RCT) and shed light on integrated treatment on palliative care in future.

## Methods

### Study design

This was a multi-centre, double-blind, randomized, placebo-controlled trial conducted at the palliative care units of three public hospitals in Hong Kong, namely Caritas Medical Centre (CMC), Our Lady of Maryknoll Hospital (OLMH) and Hong Kong Buddhist Hospital (HKBH). The study protocol had been approved by the Kowloon West Cluster [KW/FR-16-077(98-20(TCM)] and Kowloon Central/Kowloon East Cluster [KC/KE-17-0116/ER-3] of Hospital Authority Research Ethics Committees, and the Hong Kong Baptist University [HASC/15-16/0316]. It had also been registered in ClinicalTrials.gov with identifier number NCT02795390 [https://clinicaltrials.gov/ct2/show/NCT02795390] on June 10, 2016. All participants were able to give voluntary, written and informed consent on their own before entering the trial. The consent form was provided in Additional file 1. A follow-up visit was arranged to investigate the changes on bowel movement and/or any adverse effect detected after 2-week treatment.

### Participants and settings

Patients were age 18 years or above with a diagnosis of advanced cancer at the palliative care out-patient clinics of the three hospitals. They were eligible for this study if they had severity of constipation ≥ 3points (an 8-point scale from 0 = none to 7 = most severe) [5], Palliative Performance Scale (PPS) ≥ 60% (patient can be able to care for himself/herself with occasional assistance necessary), relatively stable liver and renal function (i.e. creatinine ≤ 100umol/L or creatinine clearance ≥ 60 mL/min, total bilirubin ≤ 30umol/L, transaminases ≤ 4 times above the upper limits of the normal range) within 3 months, and could read and speak Chinese. Patients were excluded if they were unable to communicate such as in cognitive impairment, presence of a colostomy or gastrointestinal obstruction, presence of loose or watery stool (Bristol stool scale 6–7) or bowel movement ≥ 3 times per day under routine laxative treatment. Patients with history of allergy to TCM, estimated life expectancy less than one month and mental incompetence in giving consent were also excluded.

### Sample size estimation

As it was a pilot study, 60 patients (30 patients per arm) were first enrolled. The results were important to calculate a sample size at a significance level of 0.025 (used to maintain the overall significance level at 5%) for a larger scale clinical trial in future.

### Randomization and blinding

All eligible subjects of both groups were randomly assigned to receive modified MZRW treatment or placebo control. For randomization, simple, complete, non-sequential random numbers were generated in advance by a computer program in a block of four and kept in sealed opaque envelopes. Two investigators with registered Chinese medicine practitioner licenses (Mok HF & Kang YC) were responsible to differentiate the TCM pattern of every patient and modify the core prescription by adding the herbs for deficiency of blood, *Yin* or *Yang*, while another two investigators (Cheng CW & Lam PY) were responsible to deliver the medication according to the codes and contact with patients. Chinese medicine practitioners (Mok HF & Kang YC) and patients were blinded in the whole study, and treatment assignments were not revealed until the termination of the whole study.

### Intervention

MZRW group (with modification): From our previous study, a TCM prescription with the functions on replenishing deficiency, redirecting the flow of *Qi* stagnation and moistening the dryness caused by the blood (*Yin*) deficiency may be helpful for advance cancer patients with constipation [9]. Therefore, MZRW granules 10 g plus *Radix Astragali* (HuangQi) granules 4 g (equivalent to 20 g raw herb) were chosen as the core prescription granules. The combined action of MZRW, which was composed of *Cannabis Fructus* (HuoMaRen) 14.4 g, Rhei Radix et Rhizoma (DaHuang) 7.2 g, Paeoniae Radix Alba (BaiShao) 3.6 g, Armeniacae Semen Amarum (KuXingRen) 7.2 g, Aurantii Fructus Immaturus (ZhiShi) 3.6 g and Magnoliae Officinalis Cortex (HouPo) 4.3 g, and *Radix Astragali* (HuangQi) 20 g could nourish *Qi*, moisten the intestine, promote the movement of *Qi*, and unblock the bowel [15]. Furthermore, six herbal granules could be added according to the pattern differentiated for individual participant. These six herbs were selected through thorough discussion among our research team. They were *Rehmanniae Radix Praeparata* (ShuDiHuang) 15 g and *Angelicae Sinensis Radix* (DangGui) 10 g for Deficiency of Blood, *Ophiopogonis Radix* (MaiDong) 15 g and *Rehmanniae Radix* (ShengDiHuang) 10 g for Deficiency of *Yin*, and *Cistanches Herba* (RouCongRong) 15 g and *Achyranthis Bidentatae Radix* (NiuXi) 10 g for Deficiency of *Yang* [16]. Adjustment on dosage was allowed for subjects with difficulties on taking research medication.

The placebos of core prescription and modifications on patterns (deficiency of blood, *Yin* and *Yang*) are made from dextrin (76.03%), tea essence (23.61%), gardenin (0.02%), and caramel (0.34%) to achieve packing, color, smell, taste, and texture comparable to the herbal granules [10]. All herbal granules and placebo were prepared by PuraPharm International (Hong Kong) according to the quality requirement listed in the Chinese Pharmacopoeia (2015 Edition) and the Registration of Proprietary Chinese Medicine of the Chinese Medicine Council of Hong Kong. The quality control reports, including test items on heavy metals and toxic elements, pesticides residues and microbial limit, were provided in Additional file 2. Patients were instructed to dissolve the granules in 150 ml of hot water, twice daily after meal for two weeks.

### Outcome measures and statistical analysis

The primary end point was the global assessment of improvement [10, 11]. The subjective feelings of adequate relief of constipation symptoms were rated by participants in comparison with their baselines. The scores from 0 to 6 represented markedly worse or better at the respective extremes. The response categories were collapsed to “improved” for score 4–6, “same” for score 3, or “worse” for score 0–2.

Secondary outcome measures included stool frequency per week, stool form assessed with 7-point Bristol Stool Scale (ranging from “separate hard lumps” to “watery”). The severity of constipation was evaluated by an 8-point scale (0 = none to 7 = most severe). Besides, individual assessment of constipation related symptoms (sensation of straining, incomplete evacuation, bloating, abdominal pain/cramping, nausea, and passing of gas) were recorded using a 7-point ordinal scale (0 = not at all and 6 = very severe) [10, 11].

All efficacy and safety analyses were conducted according to the intention-to-treat (ITT) principle. Missing values were imputed by the last-observation-carried-forward method. The statistical analysis was performed using the Statistical Packages of Social Sciences (SPSS) for Windows version 16.0. The statistical significance was defined as two-sided p-value of < 0.05. Baseline characteristics were reported as mean (SD). Baseline differences between the groups were assessed by the Student’s t-test for normally distributed continuous variables and by non-parametric Mann–Whitney U test for non-normally distributed. For categorical variables, chi-squared test or Fisher’s exact test were used. Comparisons between groups were conducted by using an analysis of covariance (ANCOVA) with baseline as covariate.

## Results

**Fig. 1:**
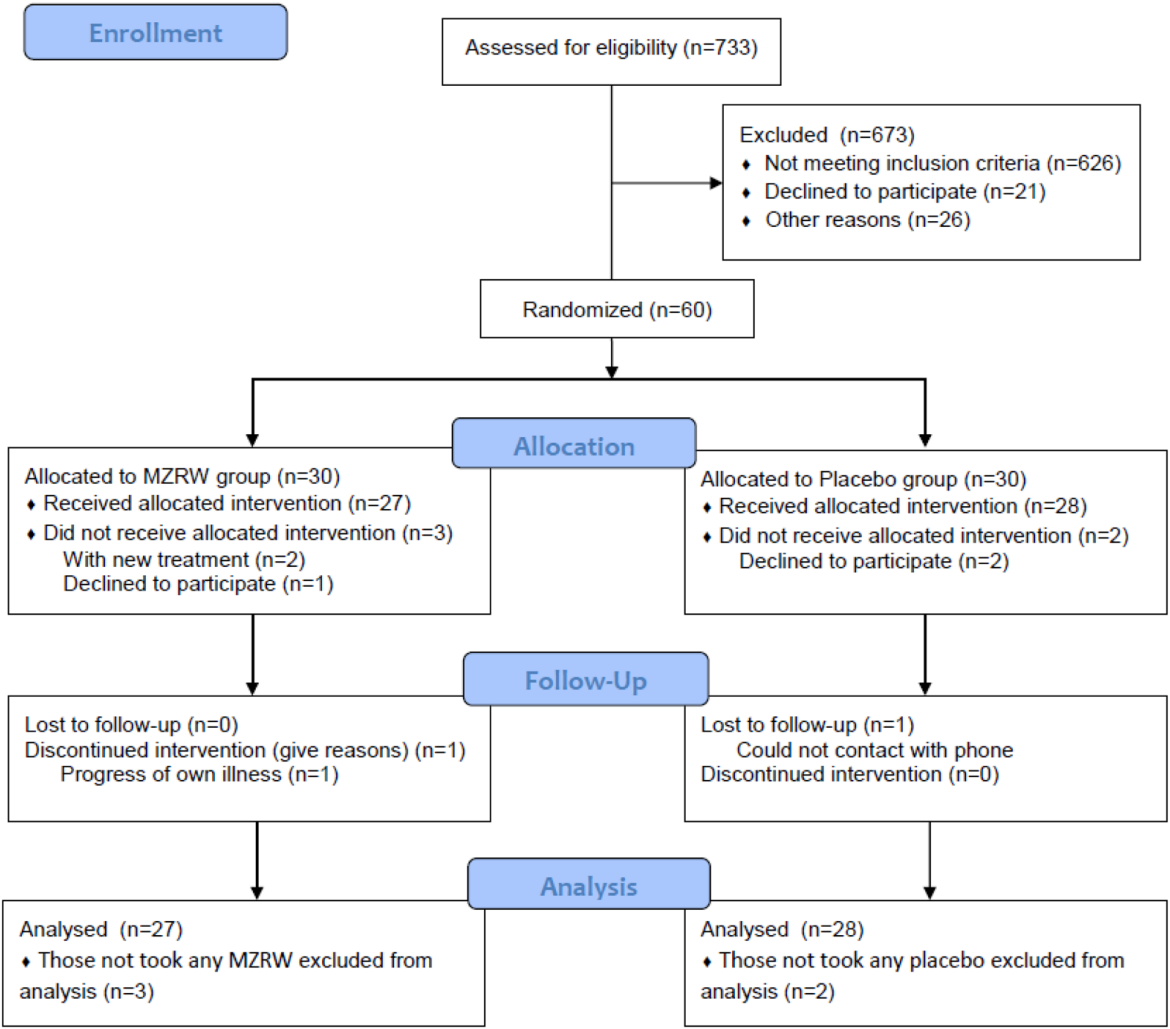
Flow diagram

From November 2016 to August 2018, 733 patients were screened. 673 patients were excluded for the following reasons: PPS < 60%, constipation severity below 3/7, colostomy done, unable to communicate, significant impairment of liver and renal function, etc. Six subjects, three from each treatment group, withdrew from the study. Five of them withdrew after randomization without receiving any research medicine. One subject had taken one dose of research medication and withdrew due to progress of her own illness. A flow diagram was shown in Fig. 1. There were no significant differences in age, gender, TCM pattern, stool frequency, stool type, severity of constipation, prescription of strong opioids and most of constipation related symptoms between the MZRW group and placebo group (Table 1).

**Table 1:** Patient demographic and baseline data

Variable	MZRW group (n = 30)	Placebo group (n = 30)	*P *value
Age, mean (SD), y	72.83 (12.24)	77.50 (10.49)	.118
Women^a^, n (%)	17 (56.67%)	12 (40.00%)	.196
TCM patterns			
Qi deficiency pattern, n (%)	21 (70.00%)	23 (76.67%)	.559
Other patterns			
Yin Deficiency, n (%)	4 (13.33%)	3 (10.00%)	NA
Yang Deficiency, n (%)	4 (13.33%)	3 (10.00%)	NA
Heat, n (%)	1 (3.33%)	0 (0)	NA
Blood Deficiency, n (%)	0 (0)	1 (3.33%)	NA
Palliative Performance Scale, mean (SD)	68.67 (8.90)	66.33 (6.69)	.256
No. of stool opening/week, mean (SD)	4.33 (2.96)	4.40 (3.82)	.940
Bristol stool type^b^, mean (SD)	2.63 (1.63)	3.17 (1.72)	.223
Severity of constipation^c^, mean (SD)	4.73 (1.19)	4.90 (1.17)	.586
Individual constipation related symptoms^d^			
Sensation of straining, mean (SD)	3.82 (1.30)	3.78 (1.40)	.924
Incomplete evacuation, mean (SD)	3.12 (1.92)	2.97 (2.11)	.775
Bloating, mean (SD)	2.23 (2.10)	2.03 (1.94)	.703
Abdominal pain/cramping, mean (SD)	1.23 (1.63)	1.33 (1.81)	.823
Nausea, mean (SD)	1.58 (1.88)	0.70 (1.42)	.044
Passing of gas, mean (SD)	2.77 (1.77)	2.87 (1.82)	.830
Prescription of strong opioids, n (%)	9 (42.86%)	8 (36.36%)	1.000

^a^*P* value based on Pearson chi−square test method

^b^Bristol stool type was measured as 1−7point ranging from “separate hard lumps” to “watery”

^c^Severity of constipation were assessed as 0−7points ranging from not at all to very severe

^d^Individual constipation related symptoms were assessed as 0−6points ranging from not at all to very severe

### Primary outcome measure

For analysing those subjects with taking at least one time of research medication, 16 subjects in the MZRW group and eight subjects in the placebo group, in total, reported improvement of their symptoms. By contrast, 10 subjects in the MZRW group and 19 subjects in the placebo group claimed that symptoms remained the same. One subject from each group reported worsening of symptoms. Hence, the global assessment of improvement was 59.3% (16/27) for the MZRW group and 28.6% (8/28) in the placebo group (p-value = 0.022). (Table 2).

**Table 2:** Comparison of treatment effect toward global assessment of improvement and other outcome measures

Variable	MZRW group (n = 27)	Placebo group (n = 28)	Between-Group Difference (95% CI)	*P *value
Primary Outcome				
Global assessment of improvement^a^, n (%)	16 (59.26%)	8 (28.57%)	NA	.022
Secondary Outcomes (Changes from baseline)				
Stool frequency, mean (SD)	2.46 (4.59)	0.45 (1.71)	2.02 (0.10 to 3.93)	.040
Bristol stool type, mean (SD)	0.89 (1.37)	0.71 (1.61)	0.17 (− 0.63 to 0.98)	.667
Severity of constipation, mean (SD)	− 1.94 (2.22)	− 0.82 (1.67)	− 1.12 (− 2.19 to -0.05)	.040
Sensation of straining, mean (SD)	− 1.09 (1.81)	− 0.02 (1.03)	− 1.07 (− 1.88 to -0.27)	.010
Incomplete evacuation, mean (SD)	− 1.41 (2.28)	− 0.46 (2.04)	− 0.94 (− 2.11 to 0.23)	.112
Bloating, mean (SD)	− 0.80 (1.97)	− 0.54 (1.91)	− 0.26 (− 1.31 to 0.79)	.621
Abdominal pain/cramping, mean (SD)	− 0.26 (1.68)	0.04 (1.43)	− 0.29 (− 1.14 to 0.55)	.485
Nausea^b^, mean (SD)	− 0.15 (1.34)	0.07 (1.76)	− 0.22 (− 1.07 to 0.63)	.606
Passing of gas, mean (SD)	− 0.33 (1.83)	− 0.43 (1.70)	0.10 (− 0.86 to 1.05)	.843

^a^P value based on Pearson chi−square test method

^b^The difference in baseline has no significant effect on nausea after treatment according to covariance analysis

### Secondary outcome measures

The mean stool frequency of the MZRW group increased by 2.46 times/week while that of placebo group increased by 0.45 times/week (p = 0.04). Further, the MZRW group had greater improvement on the score of severity of constipation and sensation of straining when comparing with that of placebo group (p-value < 0.05). There were, however, no significant differences on the changes on stool type, and sensations of incomplete evacuation, bloating, abdominal pain/cramping, nausea and passing of gas within two groups. (Table 2).

In total, 11 subjects (three in the MZRW group and eight in the placebo group) reported one or more adverse effects during the study. They included upper gastrointestinal symptoms, poor taste of research medications and changes of urination. All adverse effects were mild and subsided spontaneously. (Table 3) One subject in the MZRW group was admitted for cancer pain management.

**Table 3:** Adverse effects occurred in two groups

	TCM group (n = 27)	Placebo group (n = 28)
No. of participants with adverse effects	3	8
No. of events (Frequency)		
Stomach discomfort and/or nausea	1	3
Poor taste of medicine	0	3
Frequent or dark urine	1	2
Abdominal pain	0	1
Cough	1	0
Dizzy	0	1

## Discussion

Constipation is a common clinical problem in patients with advanced cancer but with inadequate managed. An effective medication to relieve can greatly enhance their quality of life. MZRW and its modifications is the most frequently used formulae for clinical trials on constipation based on the systematic reviews our group conducted previously [17, 18]. However, there are limited information about its effectiveness and safety for terminally ill patients. With due consideration of the generally weaker physical state among advanced cancer patients, the suggested dose of MZRW (10 g per day, 5 g b.i.d.) instead of optimal dose (15 g per day, 7.5 g b.i.d.) on functional constipation [10, 11] was prescribed with *Radix Astragali* (HuangQi). Several individual modifications for blood, *Yin* or *Yang* deficiency were also provided according to participants’ TCM patterns. In this pilot study, near 60% of the subjects reported global improvement in constipation after two-week treatment, and no serious adverse events was found. Moreover, modified MZRW is effective in increasing the bowel movements, and reducing the severity of constipation and the straining of defecation (p-value < 0.05) among palliative cancer patients. Modified MZRW is a potential treatment option for relief of constipation in advanced cancer patients. Its efficacy and acceptance among advanced cancer patients as compared with current laxatives deserve further investigation.

Opioid medications cause decrease in small and large bowel motility, increase tone in ileocaecal valve and anal sphincter. At the same time, opioid decreases electrolyte and water secretion in small bowel, and increase absorption in large bowel [19]. Therefore, opioid-induced constipation is a major subtype of secondary constipation among millions of patients with acute or chronic pains [20]. Our group has studied the pharmacology of MZRW and identified five representative active components, which can enhance colonic motility [21]. MZRW treatment in our previous study resulted in reduced levels of circulating oleamide, which is a known regulator of intestinal motility [22]. Further pharmacometabolomic analysis of MZRW for opioid-induced constipation can be explored when implementing a large-scale clinical trial in future.

Palliative care becomes a global health priority with increasing demand over the next 20 to 30 years [23, 24]. It aims to improve the quality of life of patients and their families who are facing problems associated with life-threatening illness [25]. This core idea coincides with the goal of TCM, which focuses on both wellness and quality of life [26]. TCM has long been used as a supportive intervention for cancer patients in China and many Asian countries, and is becoming popular in western counties [13]. TCM practitioners could share knowledge and experiences for mutual intellectual enrichments and shed light on integrated treatment on palliative care in future [27].

### Strengths and limitations

This pilot study was reported strictly according to the Chinese herbal medicine formula extensions of CONSORT (Consolidated Standards of Reporting Trials) Statement [28]. Certain modifications were allowed according to the subjects’ TCM patterns. We believed that the results from this project provided pragmatic clinical evidences of MZRW for advanced cancer patients with constipation in routine practice. There are three limitations to this study in drawing broad conclusion. First, the sample size was small, for which only 60 patients (30 subjects per arm) were enrolled, and no sub-group analysis on TCM patterns and prescribed medications (e.g. opioid) was made. Second, by considering the condition changes of late-stage cancer, the treatment course was relatively short and no post-treatment follow-up was provided in the study design. Third, more than one-third of patients was excluded due to PPS less than 60%, a group which is also highly susceptible to constipation. For better investigating the action of MZRW for advanced cancer patients with constipation, a fully powered RCT with longer treatment course, reasonable post treatment follow-up period, available for having multiplicity of analyses on TCM patterns and opioid intake should be carry out in future.

## Conclusion

MZRW is effective in bringing global improvement in constipation for 59.3% of patients (vs 28.6% in placebo group) with no serious adverse effects. It is more effective than placebo in increasing the number of bowel movements, and reducing the severity of constipation and the straining of defecation. MZRW is a potential treatment option for constipation in advanced cancer patients. Its long-term effectiveness and acceptance among advanced cancer patients could be further investigated in large-scale clinical trial in future.

## Supplementary Information

**Additional file 1**. Consent form.

**Additional file 2**. Quality control reports of research medications.

## Data Availability

Researchers who provide a methodologically sound proposal can request individual participant data, study protocol, statistical analysis plan and analytic code after deidentification. Proposals should be directed to bzxiang@hkbu.edu.hk to gain access.

## Abbreviations

ANCOVA: Analysis of covariance
CMC: Caritas Medical Centre
CONSORT: Consolidated Standards of Reporting Trials
ESMO: European Society for Medical Oncology
HKBH: Hong Kong Buddhist Hospital
ITT: Intention-to-treat
MZRW: MaZiRenWan
OLMH: Our Lady of Maryknoll Hospital
PPS: Palliative Performance Scale
RCT: Randomized controlled trial
SPSS: Statistical Packages of Social Sciences
TCM: Traditional Chinese medicine

## References

[1] Larkin PJ, Cherny NI, La Carpia D (2018). Diagnosis, assessment and management of constipation in advanced cancer: ESMO Clinical Practice Guidelines. Ann Oncol.

[2] Wickham RJ (2017). Managing constipation in adults with cancer. J Adv Pract Oncol.

[3] Bausewein C, Simon ST, Pralong A (2015). Palliative care of adult patients with cancer. Dtsch Arztebl Int.

[4] Laugsand EA, Jakobsen G, Kaasa S, Klepstad P (2011). Inadequate symptom control in advanced cancer patients across Europe. Support Care Cancer.

[5] Cheng CW, Kwok AO, Bian ZX, Tse DM (2012). A cross-sectional study of constipation and laxative use in advanced cancer patients: insights for revision of current practice. Support Care Cancer.

[6] Lu AP, Jia HW, Xiao C, Lu QP (2004). Theory of traditional Chinese medicine and therapeutic method of diseases. World J Gastroenterol.

[7] Jiang WY (2005). Therapeutic wisdom in traditional Chinese medicine: a perspective from modern science. Trends Pharmacol Sci.

[8] Lin YJ, Chang HT, Lin MH (2021). Professionals’ experiences and attitudes toward use of traditional Chinese medicine in hospice palliative inpatient care units: a multicenter survey in Taiwan. Integr Med Res.

[9] Cheng CW, Kwok AOL, Bian ZX, Tse DMW (2012). The quintessence of traditional Chinese medicine: syndrome and its distribution among advanced cancer patients with constipation. Evid Based Complement Alternat Med.

[10] Cheng CW, Bian ZX, Zhu LX, Wu JCY, Sung JJY (2011). Efficacy of a Chinese herbal proprietary medicine (hemp seed pill) for functional constipation. Am J Gastroenterol.

[11] Zhong LLD, Cheng CW, Kun W (2019). Efficacy of MaZiRenWan, a Chinese herbal medicine, in patients with functional constipation in a randomized controlled trial. Clin Gastroenterol Hepatol.

[12] Bharucha AE, Lacy BE (2020). Mechanisms, evaluation, and management of chronic constipation. Gastroenterology.

[13] Chung VCH, Wu X, Hui EP (2015). Effectiveness of Chinese herbal medicine for cancer palliative care: overview of systematic reviews with meta-analyses. Sci Rep.

[14] Chung VCH, Wu X, Lu P (2016). Chinese herbal medicine for symptom management in cancer palliative care: systematic review and meta-analysis. Medicine (Baltimore).

[15] Bensky D (1990). Chinese herbal medicine: formulas & strategies.

[16] Li X, Wei W (2002). Chinese materia medica: combinations and applications.

[17] Cheng CW, Bian ZX, Wu TX (2009). Systematic review of Chinese herbal medicine for functional constipation. World J Gastroenterol.

[18] Zhong LLD, Zheng G, Ge LD (2016). Chinese herbal medicine for constipation: zheng-based associations among herbs, formulae, proprietary medicines, and herb-drug interactions. Chin Med.

[19] Davies A, Leach C, Caponero R (2020). MASCC recommendations on the management of constipation in patients with advanced cancer. Support Care Cancer.

[20] Hanson B, Siddique SM, Scarlett Y, Sultan S, American Gastroenterological Association Institute Clinical Guidelines Committee (2019). American Gastroenterological Association Institute technical review on the medical management of opioid-induced constipation. Gastroenterology.

[21] Huang T, Ning ZW, Hu DD (2018). Uncovering American Gastroenterological Association Institute the mechanisms of Chinese herbal medicine (MaZiRenWan) for functional constipation by focused network pharmacology approach. Front Pharmacol.

[22] Huang T, Zhao L, Lin CY (2020). Chinese herbal medicine (MaZiRenWan) improves bowel movement in function constipation through down-regulating oleamide. Front Pharmacol.

[23] Ferris FD, Bruera E, Cherny N (2009). Palliative cancer care a decade later: accomplishments, the need, next steps—from the American Society of Clinical Oncology. J Clin Oncol.

[24] Lutz S (2011). The history of hospice and palliative care. Curr Probl Cancer.

[25] World Health Organization. Palliative care. 2020. https://www.who.int/news-room/fact-sheets/detail/palliative-care. Accessed 23 Jan 2021.

[26] Zeng S, Liu Y, Wang X (2020). Traditional Chinese medicine could play an important role in integrative palliative care in China. J Altern Complement Med.

[27] Patwardhan B, Mutalik G (2014). Search of novel model for integrative medicine. Chin J Integr Med.

[28] Cheng CW, Wu TX, Shang HC (2017). CONSORT Extension for Chinese herbal medicine formulas 2017: recommendations, explanation, and elaboration. Ann Intern Med.

