# Supplementary material for: A pilot randomized placebo-controlled study on modified MaZiRenWan: a formulated Chinese medicine to relieve constipation for palliative cancer patients
Source: Chin Med. 2022 Mar 2;17:31. doi: 10.1186/s13020-022-00580-0 (PMC8889635; doi:10.1186/s13020-022-00580-0)
Supplement: Supplementary file 1 — Additional file 1. Consent form. [file 13020_2022_580_MOESM1_ESM.doc]

**明愛醫院 聖母醫院 香港佛教醫院 紓緩治療科**

**香港浸會大學中醫藥學院 仁濟醫院-香港浸會大學中醫教研中心(西九龍)**

**香港佛教聯合會-香港大學中醫臨床教研中心**

**研究資料及知情同意書______________________________**______________________________________________________________

**題目︰針對晚期腫瘤病人便秘症狀之中西醫協作研究**

閣下獲邀參加一項針對晚期腫瘤病人便秘症狀之中西醫協作研究。在決定是否參加前，請先閱讀以下研究資料。如閣下有任何問題，請向研究人員提出，我們十分樂意為您作進一步解釋。您亦可先詢問醫生、家人及朋友的意見，再作決定。

**資料背景**

便秘是晚期腫瘤患者其中一種常見症狀，臨床上多以口服瀉藥作對症處理，但部份患者服用瀉藥仍未能緩解症狀。中藥複方同時具備調理身體，通腑消積的作用，或能更有效改善患者便秘的問題。為此，明愛醫院、聖母醫院、香港佛教醫院與香港浸會大學中醫藥學院、仁濟醫院－香港浸會大學中醫教研中心（西九龍）及香港佛教聯合會-香港大學中醫臨床教研中心進行一項針對晚期腫瘤病人便秘症狀之中西醫協作研究。

**研究目的**

中藥在臨床上治療便秘已有悠久的歷史，但鑑於現時國際認可的科研驗證不足，導致中藥的認受性普遍偏低，故此我們希望在現有治療的基礎上，透過與安慰劑作比較，證明中藥複方對晚期腫瘤病人便秘的療效。另一方面，亦可分析不同的中醫證候分型與口服瀉藥的療效關係，對推展中西醫協作治療和研究有啟導性的作用。

**研究過程**

閣下將成為六十位參與藥物臨床研究的人士之一，而參加本研究乃屬自願性質。

是次研究將會在明愛醫院、聖母醫院及香港佛教醫院紓緩治療(門診部)進行。

覆診時，閣下已獲主診醫生告知是次研究之概略。閣下隨後將被安排與研究人員會面，以確定是否符合研究要求，研究人員會向您清楚解釋整項研究的過程，而參加本研究乃屬自願性質，閣下在簽署同意書前，會給予足夠的時間去考慮是否參與這項研究，亦可隨時自由發問任何問題。

是次研究包括兩個部份。

第一部份︰中醫證候分型與口服瀉藥的療效關係

研究人員將從閣下的病歷資料了解您的身體情況及相關的檢查結果，並在研究人員協助下完成有關排便習慣的問題，藉此評估您的腸道功能。另一方面，閣下將安排會見註冊中醫師，透過以傳統中醫四診「望、聞、問、切」的方法，分析您的體質分型。

整個過程約需十至十五分鐘。

第二部份︰中草藥治療晚期腫瘤病人便秘症狀隨機對照臨床研究

若現時的治療未能緩解閣下的便秘問題，您將獲邀參加為期兩週的臨床研究。閣下所接受之藥物組別是由雙盲式電腦隨機抽樣決定，分別為(A)中藥治療組或(B)安慰劑組。完成兩週的療程後，我們會為您安排覆診，由研究人員跟進病情變化、可能出現的副作用和服藥情況等事項。

是次研究採用顆粒劑中藥，由註冊中醫師跟據閣下的體質情況處方藥物，其主要成份包括黃蓍、火麻仁、杏仁、白芍、大黃、枳實和厚朴等，達致益氣導滯，潤腸通便的目的。安慰劑則由糊精、茶精、梔子黃素、焦糖色素等食品添加劑組成。顆粒劑中藥和安慰劑從原料、生產到包裝均受到嚴格的品質監控，並合符國際優良藥品製造規範(GMP)的標準。

**潛存風險及緊急操施**

本研究藥物未有嚴重不良反應的相關報告，在臨床使用過程中或可能出現噁心、嘔吐、胃脹、腹部不適及頭暈等症狀。若閣下正接受化療、標靶治療或免疫治療等紓緩治療，服用中藥或有可能產生藥物的相互作用，如降低抗癌藥物的療效和/或增加抗癌藥物的毒性等情況。

如閣下在計劃期間感到不適，包括中藥過敏反應等，我們會即時終止閣下的研究計劃，如有需要將轉介往仁濟醫院－香港浸會大學中醫教研中心（西九龍）或香港佛教聯合會-香港大學中醫臨床教研中心作進一步治療。另一方面，參加本研究不需要抽取額外血液樣本，我們會參考閣下在服食科研中藥前和完成2週的療程後的常規化驗檢查結果，以監察肝腎功能的情況。抽血的風險包括疼痛、瘀傷、發紅、靜脈感染腫脹，及罕見的昏厥風險。本研究已投保專業責任保險，所有參加者均包括在保障範圍內。

**參加者需履行義務**

如閣下同意參加是次研究，您需向研究小組提供個人資料，包括姓名、年齡、聯絡電話等，及有關閣下健康情況的資料。而獲邀參加藥物臨床研究的人士，則需1) 按時服用科研藥物，1日2次(飯後半小時服)；2) 按預約時間返回明愛醫院、聖母醫院或香港佛教醫院紓緩治療科(門診部)覆診。

**新資料**

您將被及時告知重要的新資訊，這可能會影響您繼續參與本研究的決定。

**私隱保障**

此項研究資料可能被發表。保護閣下之私穩及保密性乃研究者之重要任務，首先問卷會以匿名形式進行，而完成的問卷將存放於上鎖之文件櫃內，並以單一順序編碼以茲識別。所有相關的資料在研究結束後會保存不少於3年，及後將全數銷毁。

根據香港法律規定（特別是第 486 章《個人資料（私隱）條例》），閣下享有或可享有確保您的個人資料保密的權利，例如在或為本研究中有關收集、監管、保留、管理、控制、使用（包括分析或比較）、轉進或轉出香港、不披露、清除和/或以任何方式處理或棄置的權利。如有任何問題，請您諮詢個人資料私隱專員或其職員（電話號碼：2827 2827），以瞭解妥善監控或監管您的個人資料保護之事宜，以確保閣下能完整掌握和瞭解遵守規管個人資料私隱的法律之重要性。

**潛在得益**

閣下不需要為研究中的任何一項操作付費。參與是次計劃除有可能幫助閣下改善便秘的症狀外，如您獲邀參加藥物臨床研究，在完成2週藥物療程後，您可於仁濟醫院－香港浸會大學中醫教研中心（西九龍）或香港佛教聯合會-香港大學中醫臨床教研中心，接受為期兩週免費的註冊中醫師內科診症及中藥治療。

**受試者之權益**

閣下參與此項研究乃自願性質，是否參加研究全視乎您的決定，即使您拒絕參加，您仍可得到您應有的醫療服務與照顧。您可以在研究期間終止參與，沒有給予理由而退出，並不會影響您現在或日後所接受的醫療及護理服務。一旦您要退出研究，如果沒有提出特別要求，銷毁退出前所收集的數據，我們將會繼續使用。如您決定終止參與是次研究或有任何問題，請致電3411 6521聯絡鄭頌華中醫師，3408 7802(明愛醫院紓緩科病房)或所屬的家訪姑娘(聖母醫院/香港佛教醫院)。

是次研究已通過九龍西和九龍中及九龍東聯網臨床研究倫理委員會的審批，閣下亦可致電九龍西(2990 1017)或九龍中及九龍東(3506 8888)聯網臨床研究倫理委員會詢問有關病人權益的問題。當簽妥參與研究同意書後，研究參加者及其合法代表授權予監測人、九龍西/九龍中及九龍東聯網臨床研究倫理委員及其他監管部門，可直接翻閱研究參加者的醫療紀錄正本，以查核研究進行的程序，及/或資料，而不違反根據相關法律及指引所確保研究參加者個人資料保密之權利。

**明愛醫院 聖母醫院 香港佛教醫院 紓緩治療科**

**香港浸會大學中醫藥學院 仁濟醫院-香港浸會大學中醫教研中心(西九龍)**

**香港佛教聯合會-香港大學中醫臨床教研中心**

**研究資料及知情同意書**

**___________________________________________________________________**

**題目︰針對晚期腫瘤病人便秘症狀之中西醫協作研究**

本人已閱讀此份知情同意書之內容 (或已被講解此份知情同意書之內容)。

本人已有機會提出及討論疑問，而提出的所有問題已得到滿意的答覆。

本人同意參與這項研究，而參與是項研究純粹出於自願性質。

本人明白本人將會獲發已簽署的書面知情同意書的副本。

_________________________________ _________________________

病人簽署 日期

_________________________________

病人姓名 (正楷)

_________________________________ _________________________

見證者簽署 日期

_________________________________

見證者姓名 (正楷)

_________________________________ _________________________

研究者簽署 日期

_________________________________

研究者姓名 (正楷)
