# Supplementary material for: A pilot randomized placebo-controlled study on modified MaZiRenWan: a formulated Chinese medicine to relieve constipation for palliative cancer patients
Source: Chin Med. 2022 Mar 2;17:31. doi: 10.1186/s13020-022-00580-0 (PMC8889635; doi:10.1186/s13020-022-00580-0)
Supplement: Supplementary file 2 — Additional file 2. Quality control reports of research medications. [file 13020_2022_580_MOESM2_ESM.pdf]

培力（南寧）藥業有限公司檢測中心 **PuraPharm (Nanning) Pharmaceuticals Co.,Ltd. Testing Laboratory**
中國廣西南寧市高新技術開發區科園大道 46 號
No.46, Ke Yuan Road,Nanning New & High-tech Industrial Development Zone, Guangxi,China.

對“麻子仁丸配方顆粒”之樣品之分析報告

Report on the submitted sample identified by the client - Ma Zi Ren Wan

樣品名稱 Product Description	麻子仁丸配方顆粒 Ma Zi Ren Wan
樣品編號 Product Code	2173
樣品規格 Product Specification	200 克/瓶 g/ bottle
批號 Batch No.	A1600682
有效期至(年/月/日)Expiry Date（yyyy/mm/dd）	2019/05/18
本批數量 Quantity Produced	3968 瓶 bottles
樣品收到時狀態 Sample Receiving Condition	室溫下存放於密封塑膠樽原來包裝中<br>In sealed bottle of the original package under ambient condition.
製造商 Manufacturer	生產部 Production Department
委託檢驗單位 Inspected Entity	生產部 Production Department
來源地 Region of Origin	生產部 Production Department
目的地 Region of Destination	培力（南寧）藥業有限公司檢測中心<br>PuraPharm (Nanning) Pharmaceuticals Co.,Ltd. Testing Laboratory
檢驗日期(年/月/日)Testing Period（yyyy/mm/dd）	2016/05/24 - 2016/05/30

測試項目、分析方法及分析結果 Test Requested, Test Method and Test Results

請參考續頁。Please refer to the following page(s)

****

培力（南寧）藥業有限公司檢測中心代表簽名

Signed for and on behalf of PuraPharm (Nanning) Pharmaceuticals Co.,Ltd. Testing Laboratory

測試項目及分析方法 Test Requested and Test Method

測試項目 Test Items	參考方法 Reference Method
1. 性狀 Appearance	中國藥典, 2015 年版, 第四部, 通則 0104<br>The Pharmacopoeia of the People's Republic of China 2015, Vol.4, General chapter 0104
2. 水分 Determination of Water	中國藥典, 2015 年版, 第四部, 通則 0832<br>The Pharmacopoeia of the People's Republic of China 2015, Vol.4, General chapter 0832
3. 粒度 Size of Granule	中國藥典, 2015 年版, 第四部, 通則 0982<br>The Pharmacopoeia of the People's Republic of China 2015, Vol.4, General chapter 0982
4. 溶化性<br>Determination of Dispersibility	中國藥典, 2015 年版, 第四部, 通則 0104<br>The Pharmacopoeia of the People's Republic of China 2015, Vol.4, General chapter 0104
5. 裝量 Capacity	中國藥典, 2015 年版, 第四部, 通則 0942<br>The Pharmacopoeia of the People's Republic of China 2015, Vol.4, General chapter 0942
6. 重金屬及有害元素<br>Heavy Metals and Toxic Elements	中國藥典、2015 年版, 第四部, 通則 2321；電感耦合等離子體質譜法；<br>The Pharmacopoeia of the People's Republic of China 2015, Vol.4, General chapter 2321；ICP-MS
7. 農藥殘留 Pesticides Residues	香港中醫藥管理委員會《中成藥註冊申請手冊》<br>Chinese Medicine Council of Hong Kong《Application Form: Registration of proprietary Chinese medicines》
8. 微生物限度 Microbial Limit	中國藥典、2015 年版, 第四部, 通則 1105、1106、1107<br>The Pharmacopoeia of the People's Republic of China 2015, Vol.4, General chapter 1105，1106，1107
- 需氧菌總數 TAMC (Total Aerobic Microbial Count)	
- 霉菌和酵母菌總數 TYMC (Total Yeast and Mold Microbial Count)	
- 大腸埃希菌 Escherichia Coli	

****

編制 Report compiled by：[signature]　　審核 Reviewed by：[signature]

分析結果 Test Results

測試項目 Test Items	品質標準 Quality Specification	麻子仁丸配方顆粒 Ma Zi Ren Wan
1. 性狀 Appearance	本品為灰黃色至黃褐色顆粒，氣香，味辛，微苦。The product is greyish yellow to yellowish brown granule, aromatic odour, pungent with slightly bitter taste.	符合規定 Conform
2. 水分 Determination of Water	≤6.5% (W/W)	4.9%<br>符合規定 Conform
3. 粒度 Size of Granule	不能通過一號篩和能通過五號篩的顆粒和粉末總和不得超過 15% Sum of weight of granules that cannot pass through sieve No.1 and weight of powder that can pass through sieve No.5≤15%	5%<br>符合規定 Conform
4. 溶化性<br>Determination of Dispersibility	應符合規定 Shall meet the requirement	符合規定 Conform
5. 裝量 Capacity	應符合規定 Shall meet the requirement	符合規定 Conform
6. 重金屬及有害元素#<br>Heavy Metals and Toxic Elements		
- 銅 Copper (Cu)	不得過 150.00mg/kg<br>Cu≤150.00 mg/kg	3.760mg/kg<br>符合規定 Conform
- 砷 Arsenic (As)	不得過 41.67mg/kg 或 1500μg/日<br>As≤41.67mg/kg or 1,500μg/day	0.084mg/kg（3.02μg/日 day）<br>符合規定 Conform
- 鎘 Cadmium (Cd)	不得過 97.22mg/kg 或 3500μg/劑<br>Cd≤97.22mg/kg or 3,500μg/dose	0.008mg/kg（0.29μg/劑 dose）<br>符合規定 Conform
- 鉛 Lead (Pb)	不得過 4.97mg/kg 或 179μg/日<br>Pb≤4.97mg/kg or 179μg/day	0.059mg/kg（2.12μg/日 day）<br>符合規定 Conform
- 汞 Mercury (Hg)	不得過 1.00mg/kg 或 36μg/日<br>Hg≤1.00mg/kg or 36μg/day	＜0.001mg/kg（＜0.04μg/日 day）<br>符合規定 Conform
7. 農藥殘留#<br>Pesticides Residues		
- 艾氏劑及狄氏劑<br>Aldrin & Dieldrin	艾氏劑及狄氏劑兩者之和≤0.05 毫克/千克<br>Sum of aldrin & dieldrin≤0.05 mg/kg<br>（檢出限艾氏劑 1.77μg/kg,狄氏劑 2.43μg/kg<br>LOD aldrin 1.77μg/kg, dieldrin 2.43μg/kg）	未檢出 Not detected<br>符合規定 Conform
- 氯丹 Chlordane	順式-,反式及氧化氯丹之和≤0.05 毫克/千克<br>Sum of cis-,trans- & oxy-chlordane≤0.05 mg/kg<br>（檢出限順式氯丹 2.05μg/kg, 反式氯丹 1.81μg/kg, 氧化氯丹 2.21μg/kg<br>LOD cis- chlordane 2.05μg/kg, trans- chlordane 1.81μg/kg, oxy-chlordane 2.21μg/kg）	未檢出 Not detected<br>符合規定 Conform
- 滴滴涕 DDT	4,4'-滴滴涕, 2,4'-滴滴涕, 4,4'-滴滴伊及 4,4'-滴滴滴之和≤1.0 毫克/千克<br>Sum of *p, p'*-DDT, *o,p'*-DDT, *p,p'*-DDE, *p,p'*-TDE ≤1.0 mg/kg<br>（檢出限 4,4'-滴滴涕 3.73μg/kg, 2,4'-滴滴涕 4.48μg/kg, 4,4'-滴滴伊 2.29μg/kg, 4,4'-滴滴滴 2.88μg/kg<br>LOD *p, p'*-DDT 3.73μg/kg, *o,p'*-DDT 4.48μg/kg, *p,p'*-DDE 2.29μg/kg, *p,p'*-TDE 2.88μg/kg）	未檢出 Not detected<br>符合規定 Conform
- 異狄氏劑 Endrin	≤0.05 mg/kg<br>（檢出限 LOD 3.78μg/kg）	未檢出 Not detected<br>符合規定 Conform

****

編制 Report compiled by：[signature] 審核 Reviewed by：[signature]

培力（南寧）藥業有限公司檢測中心 PuraPharm (Nanning) Pharmaceuticals Co.,Ltd. Testing Laboratory
郵編(Postal No.)：530007 電話(Tel)：0771-3218026 傳真(Fax)：0771-3216602

- 七氯 Heptachlor	七氯及環氧七氯之和≤0.05 毫克/千克<br>Sum of heptachlor & heptachlor epoxide≤0.05 mg/kg<br>（檢出限七氯 1.78μg/kg, 環氧七氯 1.97μg/kg<br>LOD heptachlor 1.78μg/kg, heptachlor epoxide 1.97μg/kg）	未檢出 Not detected<br>符合規定 Conform
- 六氯苯 Hexachlorobenzene	≤0.1 mg/kg<br>（檢出限 LOD 2.16μg/kg）	未檢出 Not detected<br>符合規定 Conform
- 六六六 Hexachlorocyclohexane	α-,β-及 δ-異構體之和≤0.3 毫克/千克<br>Sum of α-,β-,δ- isomers≤0.3 mg/kg<br>（檢出限 α-六六六 1.61μg/kg, β-六六六 2.96μg/kg, δ-六六六 1.43μg/kg<br>LOD α- HCH 1.61μg/kg, β- HCH 2.96μg/kg, δ- HCH 1.43μg/kg）	3.44×10^{-3}mg/kg<br>符合規定 Conform
- 林丹 Lindane	≤0.6 mg/kg<br>（檢出限 LOD 1.56μg/kg）	未檢出 Not detected<br>符合規定 Conform
- 五氯硝基苯 Quintozene	五氯硝基苯，五氯苯胺及甲基五氯硫基苯之和≤1.0 毫克/千克<br>Sum of quintozene, pentachloroaniline and methyl pentachlorophenyl sulphide≤1.0 mg/kg<br>（檢出限五氯硝基苯 1.92μg/kg, 五氯苯胺 1.59μg/kg, 甲基五氯硫基苯 1.33μg/kg<br>LOD quintozene 1.92μg/kg , pentachloroaniline 1.59μg/kg , methyl pentachlorophenyl sulphide 1.33μg/kg）	未檢出 Not detected<br>符合規定 Conform
8. 微生物限度#<br>Microbial Limit		
- 需氧菌總數 TAMC (Total Aerobic Microbial Count)	≤500cfu/g	＜10cfu/g<br>符合規定 Conform
-霉菌和酵母菌總數 TYMC (Total Yeast and Mold Microbial Count)	≤100cfu/g	＜10cfu/g<br>符合規定 Conform
- 大腸埃希菌 Escherichia Coli	不得檢出/克 Not detected/g	未檢出/克 Not detected/g<br>符合規定 Conform

****

編制 Report compiled by：[signature]　　審核 Reviewed by：[signature]

培力（南寧）藥業有限公司檢測中心 PuraPharm (Nanning) Pharmaceuticals Co.,Ltd. Testing Laboratory
郵編(Postal No.)：530007　電話(Tel)：0771-3218026　傳真(Fax)：0771-3216602

註：Remarks

1、 報告無“檢驗/檢測報告專用章”和缺騎縫章無效。

Test report without the stamp of “For Test/Test Report Only” is not authorized.

2、 複製的報告未重新加蓋“檢驗/檢測報告專用章”及騎縫章無效。

Certified copy of test reportwithout the stamp of“For Test/Test Report Only” is not authorized.

3、 報告無編制、審核及本中心代表簽字無效。

Test report must be authorized by the signature of the bodies of preparation, verification and representative of the testing centre.

4、 報告塗改、缺頁無效。

Test report with modification or lacking of page(s) is not authorized.

5、 對報告若有異議，請於收到報告之日起十五日內向檢驗單位提出書面申訴，否則按認可檢驗報告處理。本中心異議受理電話 0771-3218026。

The client is aggrieved by the test result. A request for review shall state in writing the reasons relied upon and shall be made to the testing centre within 15 days after receipt of the authorized test report. No requirement will be accepted after the definite time. Inquiry hot line is 0771-3218026.

6、 送樣委託檢驗，樣品名稱為委託單位自報名稱，報告僅對來樣負責。部份複製檢驗/檢測報告無效。

Name of tested product is provided by the client. The report will refer only to the sample tested. Copy of partial of the test report is not authorized.

# - 其他微生物限度要求: Other requirements for Microbial Limit Test

1. 含動物類藥材（包括提取物）的中藥固體製劑，每 10g 不得檢出沙門菌。

TCM solid preparation containing animal raw materials (including extract): Absence of Salmonella (10g).

2. 含藥材原粉的中藥固體製劑，每 10g 不得檢出沙門菌；耐膽鹽革蘭陰性菌應小於 10^2cfu（1g）。

TCM solid preparation containing herbal raw powders: Absence of Salmonella (10g); Not more than 10^2cfu of Bile-tolerant gram-negative bacteria (1g).

*** End of Report 報告完 ***

培力（南寧）藥業有限公司檢測中心 PuraPharm (Nanning) Pharmaceuticals Co.,Ltd. Testing Laboratory
郵編(Postal No.)：530007　　電話(Tel)：0771-3218026　　傳真(Fax)：0771-3216602

培力（南寧）藥業有限公司檢測中心 **PuraPharm (Nanning) Pharmaceuticals Co.,Ltd. Testing Laboratory**

中國廣西南寧市高新技術開發區科園大道 46 號

No.46, Ke Yuan Road,Nanning New & High-tech Industrial Development Zone, Guangxi,China.

對“黃芪配方顆粒”之樣品之分析報告

Report on the submitted sample identified by the client - Huang Qi

樣品名稱 Product Description	黃芪配方顆粒 Huang Qi
樣品編號 Product Code	1008
樣品規格 Product Specification	200 克/瓶 g/ bottle
批號 Batch No.	A1600806
有效期至(年/月/日)Expiry Date（yyyy/mm/dd）	2019/06/21
本批數量 Quantity Produced	25374 瓶 bottles
樣品收到時狀態 Sample Receiving Condition	室溫下存放於密封塑膠樽原來包裝中<br>In sealed bottle of the original package under ambient condition.
製造商 Manufacturer	生產部 Production Department
委託檢驗單位 Inspected Entity	生產部 Production Department
來源地 Region of Origin	生產部 Production Department
目的地 Region of Destination	培力（南寧）藥業有限公司檢測中心<br>PuraPharm (Nanning) Pharmaceuticals Co.,Ltd. Testing Laboratory
檢驗日期(年/月/日)Testing Period（yyyy/mm/dd）	2016/07/22 - 2016/07/28

測試項目、分析方法及分析結果 Test Requested, Test Method and Test Results

請參考續頁。Please refer to the following page(s)

****

培力（南寧）藥業有限公司檢測中心代表簽名

Signed for and on behalf of PuraPharm (Nanning) Pharmaceuticals Co.,Ltd. Testing Laboratory

測試項目及分析方法 Test Requested and Test Method

測試項目 Test Items	参考方法 Reference Method
1. 性狀 Appearance	中國藥典, 2015 年版, 第四部, 通則 0104<br>The Pharmacopoeia of the People's Republic of China 2015, Vol.4, General chapter 0104
2. 鑒別（1） Identification（1）	中國藥典, 2015 年版, 第四部, 通則 0502<br>The Pharmacopoeia of the People's Republic of China 2015, Vol.4, General chapter 0502
3. 鑒別（2） Identification（2）	
4. 浸出物 Extractives	中國藥典, 2015 年版, 第四部, 通則 2201<br>The Pharmacopoeia of the People's Republic of China 2015, Vol.4, General chapter 2201
5. 水分 Determination of Water	中國藥典, 2015 年版, 第四部, 通則 0832<br>The Pharmacopoeia of the People's Republic of China 2015, Vol.4, General chapter 0832
6. 粒度 Size of Granule	中國藥典, 2015 年版, 第四部, 通則 0982<br>The Pharmacopoeia of the People's Republic of China 2015, Vol.4, General chapter 0982
7. 溶化性<br>Determination of Dispersibility	中國藥典, 2015 年版, 第四部, 通則 0104<br>The Pharmacopoeia of the People's Republic of China 2015, Vol.4, General chapter 0104
8. 裝量 Capacity	中國藥典, 2015 年版, 第四部, 通則 0942<br>The Pharmacopoeia of the People's Republic of China 2015, Vol.4, General chapter 0942
9. 重金屬及有害元素<br>Heavy Metals and Toxic Elements	中國藥典, 2015 年版, 第四部, 通則 2321；電感耦合等離子體質譜法；<br>The Pharmacopoeia of the People's Republic of China 2015, Vol.4, General chapter 2321；ICP-MS
10. 農藥殘留 Pesticides Residues	香港中醫藥管理委員會《中成藥註冊申請手冊》<br>Chinese Medicine Council of Hong Kong《Application Form: Registration of proprietary Chinese medicines》
11. 微生物限度 Microbial Limit	中國藥典, 2015 年版, 第四部, 通則 1105、1106、1107<br>The Pharmacopoeia of the People's Republic of China 2015, Vol.4, General chapter 1105，1106，1107
- 需氧菌總數 TAMC (Total Aerobic Microbial Count)	
- 霉菌和酵母菌總數 TYMC (Total Yeast and Mold Microbial Count)	
- 大腸埃希菌 Escherichia Coli	
- 沙門菌 Salmonella	
- 金黃色葡萄球菌<br>Staphylococcus Aureus	
12. 含量測定 Assay	中國藥典, 2015 年版, 第四部, 通則 0512<br>The Pharmacopoeia of the People's Republic of China 2015, Vol.4, General chapter 0512

****

編制 Report compiled by： 審核 Reviewed by：

培力（南寧）藥業有限公司檢測中心 PuraPharm (Nanning) Pharmaceuticals Co.,Ltd. Testing Laboratory
郵編(Postal No.)：530007 電話(Tel)：0771-3218026 傳真(Fax)：0771-3216602

分析結果 Test Results

測試項目 Test Items	品質標準 Quality Specification	黃芪配方顆粒 Huang Qi
1. 性狀 Appearance	本品為淺黃色至棕黃色顆粒；氣微，味微甜。 The product is light yellow to yellowish brown granule, slight odour, slightly sweet taste.	符合規定 Conform
2. 鑒別（1） Identification（1）	應符合規定 Shall meet the requirement	符合規定 Conform
3. 鑒別（2） Identification（2）	應符合規定 Shall meet the requirement	符合規定 Conform
4. 浸出物 Extractives	不得少於 14.0% No less than 14.0%	21.9% 符合規定 Conform
5. 水分 Determination of Water	≤6.5% (W/W)	4.3% 符合規定 Conform
6. 粒度 Size of Granule	不能通過一號篩和能通過五號篩的顆粒和粉末總和不得超過 15% Sum of weight of granules that cannot pass through sieve No.1 and weight of powder that can pass through sieve No.5≤15%	3% 符合規定 Conform
7. 溶化性 Determination of Dispersibility	應符合規定 Shall meet the requirement	符合規定 Conform
8. 裝量 Capacity	應符合規定 Shall meet the requirement	符合規定 Conform
9. 重金屬及有害元素# Heavy Metals and Toxic Elements		
- 銅 Copper (Cu)	不得過 30.00mg/kg Cu≤30.00 mg/kg	0.866mg/kg 符合規定 Conform
- 砷 Arsenic (As)	不得過 3mg/kg As≤3mg/kg	0.191mg/kg 符合規定 Conform
- 鎘 Cadmium (Cd)	不得過 0.4mg/kg Cd≤0.4mg/kg	0.007mg/kg 符合規定 Conform
- 鉛 Lead (Pb)	不得過 8mg/kg Pb≤8mg/kg	0.060mg/kg 符合規定 Conform
- 汞 Mercury (Hg)	不得過 0.3mg/kg Hg≤0.3mg/kg	＜0.001mg/kg 符合規定 Conform
10. 農藥殘留# Pesticides Residues		
- 艾氏劑及狄氏劑 Aldrin & Dieldrin	艾氏劑及狄氏劑兩者之和≤0.05 毫克/千克 Sum of aldrin & dieldrin≤0.05 mg/kg （檢出限艾氏劑 1.77μg/kg,狄氏劑 2.43μg/kg LOD aldrin 1.77μg/kg, dieldrin 2.43μg/kg）	未檢出 Not detected 符合規定 Conform
- 氯丹 Chlordane	順式-,反式及氧化氯丹之和≤0.05 毫克/千克 Sum of cis-,trans- & oxy-chlordane≤0.05 mg/kg （檢出限順式氯丹 2.05μg/kg, 反式氯丹 1.81μg/kg, 氧化氯丹 2.21μg/kg LOD cis- chlordane 2.05μg/kg, trans- chlordane 1.81μg/kg, oxy-chlordane 2.21μg/kg）	未檢出 Not detected 符合規定 Conform
- 滴滴涕 DDT	4,4'-滴滴涕, 2,4'-滴滴涕, 4,4'-滴滴伊及 4,4'-滴滴滴之和≤1.0 毫克/千克 Sum of *p, p*'-DDT, *o,p*'-DDT, *p,p*'-DDE, *p,p*'-TDE ≤1.0 mg/kg （檢出限 4,4'-滴滴涕 3.73μg/kg, 2,4'-滴滴涕 4.48μg/kg, 4,4'-滴滴伊 2.29μg/kg, 4,4'-滴滴滴 2.88μg/kg LOD *p, p*'-DDT 3.73μg/kg, *o,p*'-DDT 4.48μg/kg, *p,p*'-DDE 2.29μg/kg, *p,p*'-TDE 2.88μg/kg）	未檢出 Not detected 符合規定 Conform
- 異狄氏劑 Endrin	≤0.05 mg/kg （檢出限 LOD 3.78μg/kg）	未檢出 Not detected 符合規定 Conform

****

編制 Report compiled by：[signature]　　審核 Reviewed by：[signature]

培力（南寧）藥業有限公司檢測中心 PuraPharm (Nanning) Pharmaceuticals Co.,Ltd. Testing Laboratory
郵編(Postal No.)：530007　　電話(Tel)：0771-3218026　　傳真(Fax)：0771-3216602

- 七氯 Heptachlor	七氯及環氧七氯之和≤0.05 毫克/千克<br>Sum of heptachlor & heptachlor epoxide≤0.05 mg/kg<br>（檢出限七氯 1.78μg/kg, 環氧七氯 1.97μg/kg<br>LOD heptachlor 1.78μg/kg, heptachlor epoxide 1.97μg/kg）	未檢出 Not detected<br>符合規定 Conform
- 六氯苯 Hexachlorobenzene	≤0.1 mg/kg<br>（檢出限 LOD 2.16μg/kg）	未檢出 Not detected<br>符合規定 Conform
- 六六六 Hexachlorocyclohexane	α-,β-及 δ-異構體之和≤0.3 毫克/千克<br>Sum of α-,β-,δ- isomers≤0.3 mg/kg<br>（檢出限 α-六六六 1.61μg/kg, β-六六六 2.96μg/kg, δ-六六六 1.43μg/kg<br>LOD α- HCH 1.61μg/kg, β- HCH 2.96μg/kg, δ- HCH 1.43μg/kg）	未檢出 Not detected<br>符合規定 Conform
- 林丹 Lindane	≤0.6 mg/kg<br>（檢出限 LOD 1.56μg/kg）	未檢出 Not detected<br>符合規定 Conform
- 五氯硝基苯 Quintozene	五氯硝基苯，五氯苯胺及甲基五氯硫基苯之和≤1.0 毫克/千克<br>Sum of quintozene, pentachloroaniline and methyl pentachlorophenyl sulphide≤1.0 mg/kg<br>（檢出限五氯硝基苯 1.92μg/kg, 五氯苯胺 1.59μg/kg, 甲基五氯硫基苯 1.33μg/kg<br>LOD quintozene 1.92μg/kg , pentachloroaniline 1.59μg/kg , methyl pentachlorophenyl sulphide 1.33μg/kg）	未檢出 Not detected<br>符合規定 Conform
11. 微生物限度#<br>Microbial Limit		
- 需氧菌總數 TAMC (Total Aerobic Microbial Count)	≤500cfu/g	10cfu/g<br>符合規定 Conform
-霉菌和酵母菌總數 TYMC (Total Yeast and Mold Microbial Count)	≤100cfu/g	＜10cfu/g<br>符合規定 Conform
- 大腸埃希菌 Escherichia Coli	不得檢出/克 Not detected/g	未檢出/克 Not detected/g<br>符合規定 Conform
- 沙門菌 Salmonella	不得檢出/10 克 Not detected/10g	未檢出/10 克 Not detected/10g<br>符合規定 Conform
- 金黃色葡萄球菌 Staphylococcus Aureus	不得檢出/克 Not detected/g	未檢出/克 Not detected/g<br>符合規定 Conform
12. 含量測定 Assay	含黃芪甲苷不得低於 0.30mg/g<br>Astragaloside IV no less than 0.30mg/g	1.03mg/g<br>符合規定 Conform
	含毛蕊異黃酮葡萄糖苷不得低於 0.24mg/g<br>Calycosin7-O-β-D-Glucopyranoside no less than 0.24mg/g	0.29mg/g<br>符合規定 Conform

****

編制 Report compiled by：[signature] 審核 Reviewed by：[signature]

培力（南寧）藥業有限公司檢測中心 PuraPharm (Nanning) Pharmaceuticals Co.,Ltd. Testing Laboratory
郵編(Postal No.)：530007 電話(Tel)：0771-3218026 傳真(Fax)：0771-3216602

註：Remarks

1、 報告無“檢驗/檢測報告專用章”和缺騎縫章無效。

Test report without the stamp of "For Test/Test Report Only" is not authorized.

2、 複製的報告未重新加蓋“檢驗/檢測報告專用章”及騎縫章無效。

Certified copy of test reportwithout the stamp of"For Test/Test Report Only" is not authorized.

3、 報告無編制、審核及本中心代表簽字無效。

Test report must be authorized by the signature of the bodies of preparation, verification and representative of the testing centre.

4、 報告塗改、缺頁無效。

Test report with modification or lacking of page(s) is not authorized.

5、 對報告若有異議，請於收到報告之日起十五日内向檢驗單位提出書面申訴，否則按認可檢驗報告處理。本中心異議受理電話 0771-3218026。

The client is aggrieved by the test result. A request for review shall state in writing the reasons relied upon and shall be made to the testing centre within 15 days after receipt of the authorized test report. No requirement will be accepted after the definite time. Inquiry hot line is 0771-3218026.

6、 送樣委託檢驗，樣品名稱為委託單位自報名稱，報告僅對來樣負責。部份複製檢驗/檢測報告無效。

Name of tested product is provided by the client. The report will refer only to the sample tested. Copy of partial of the test report is not authorized.

# - 其他微生物限度要求: Other requirements for Microbial Limit Test

1. 含動物類藥材（包括提取物）的中藥固體製劑，每 10g 不得檢出沙門菌。

TCM solid preparation containing animal raw materials (including extract): Absence of Salmonella (10g).

2. 含藥材原粉的中藥固體製劑，每 10g 不得檢出沙門菌；耐膽鹽革蘭陰性菌應小於 10^2cfu（1g）。

TCM solid preparation containing herbal raw powders: Absence of Salmonella (10g); Not more than 10^2cfu of Bile-tolerant gram-negative bacteria (1g).

*** End of Report 報告完 ***

培力（南寧）藥業有限公司檢測中心 PuraPharm (Nanning) Pharmaceuticals Co.,Ltd. Testing Laboratory

郵編(Postal No.)：530007　　電話(Tel)：0771-3218026　　傳真(Fax)：0771-3216602

培力（南寧）藥業有限公司檢測中心 **PuraPharm (Nanning) Pharmaceuticals Co.,Ltd. Testing Laboratory**
中國廣西南寧市高新技術開發區科園大道 46 號
No.46, Ke Yuan Road,Nanning New & High-tech Industrial Development Zone, Guangxi,China.

對 "熟地黃配方顆粒" 之樣品之分析報告

Report on the submitted sample identified by the client - Shu Di Huang

樣品名稱 Product Description	熟地黃配方顆粒 Shu Di Huang
樣品編號 Product Code	1031
樣品規格 Product Specification	200 克/瓶 g/ bottle
批號 Batch No.	A1600584
有效期至(年/月/日)Expiry Date（yyyy/mm/dd）	2019/04/29
本批數量 Quantity Produced	9964 瓶 bottles
樣品收到時狀態 Sample Receiving Condition	室溫下存放於密封塑膠樽原來包裝中<br>In sealed bottle of the original package under ambient condition.
製造商 Manufacturer	生產部 Production Department
委託檢驗單位 Inspected Entity	生產部 Production Department
來源地 Region of Origin	生產部 Production Department
目的地 Region of Destination	培力（南寧）藥業有限公司檢測中心<br>PuraPharm (Nanning) Pharmaceuticals Co.,Ltd. Testing Laboratory
檢驗日期(年/月/日)Testing Period（yyyy/mm/dd）	2016/05/06 - 2016/05/11

測試項目、分析方法及分析結果 Test Requested, Test Method and Test Results

請參考續頁。Please refer to the following page(s)

****

培力（南寧）藥業有限公司檢測中心代表簽名

Signed for and on behalf of PuraPharm (Nanning) Pharmaceuticals Co.,Ltd. Testing Laboratory

測試項目及分析方法 Test Requested and Test Method

測試項目 Test Items	參考方法 Reference Method
1. 性狀 Appearance	中國藥典, 2015 年版, 第四部, 通則 0104<br>The Pharmacopoeia of the People's Republic of China 2015, Vol.4, General chapter 0104
2. 水分 Determination of Water	中國藥典, 2015 年版, 第四部, 通則 0832<br>The Pharmacopoeia of the People's Republic of China 2015, Vol.4, General chapter 0832
3. 粒度 Size of Granule	中國藥典, 2015 年版, 第四部, 通則 0982<br>The Pharmacopoeia of the People's Republic of China 2015, Vol.4, General chapter 0982
4. 溶化性<br>Determination of Dispersibility	中國藥典, 2015 年版, 第四部, 通則 0104<br>The Pharmacopoeia of the People's Republic of China 2015, Vol.4, General chapter 0104
5. 裝量 Capacity	中國藥典, 2015 年版, 第四部, 通則 0942<br>The Pharmacopoeia of the People's Republic of China 2015, Vol.4, General chapter 0942
6. 重金屬及有害元素<br>Heavy Metals and Toxic Elements	中國藥典, 2015 年版, 第四部, 通則 2321；電感耦合等離子體質譜法；<br>The Pharmacopoeia of the People's Republic of China 2015, Vol.4, General chapter 2321；ICP-MS
7. 農藥殘留 Pesticides Residues	香港中醫藥管理委員會《中成藥註冊申請手冊》<br>Chinese Medicine Council of Hong Kong《Application Form: Registration of proprietary Chinese medicines》
8. 微生物限度 Microbial Limit	中國藥典, 2015 年版, 第四部, 通則 1105、1106、1107<br>The Pharmacopoeia of the People's Republic of China 2015, Vol.4, General chapter 1105，1106，1107
- 需氧菌總數 TAMC (Total Aerobic Microbial Count)	
- 霉菌和酵母菌總數 TYMC (Total Yeast and Mold Microbial Count)	
- 大腸埃希菌 Escherichia Coli	

****

編制 Report compiled by： 審核 Reviewed by：

培力（南寧）藥業有限公司檢測中心 PuraPharm (Nanning) Pharmaceuticals Co.,Ltd. Testing Laboratory
郵編(Postal No.)：530007 電話(Tel)：0771-3218026 傳真(Fax)：0771-3216602

分析結果 Test Results

測試項目 Test Items	品質標準 Quality Specification	熟地黃配方顆粒 Shu Di Huang
1. 性狀 Appearance	本品為灰黃色至黑褐色顆粒，氣微，味甜。The product is greyish yellow to dark brown granule, slight odour, sweet taste.	符合規定 Conform
2. 水分 Determination of Water	≤6.5% (W/W)	3.6%<br>符合規定 Conform
3. 粒度 Size of Granule	不能通過一號篩和能通過五號篩的顆粒和粉末總和不得超過 15% Sum of weight of granules that cannot pass through sieve No.1 and weight of powder that can pass through sieve No.5≤15%	2%<br>符合規定 Conform
4. 溶化性 Determination of Dispersibility	應符合規定 Shall meet the requirement	符合規定 Conform
5. 裝量 Capacity	應符合規定 Shall meet the requirement	符合規定 Conform
6. 重金屬及有害元素# Heavy Metals and Toxic Elements		
- 銅 Copper (Cu)	不得過 150.00mg/kg<br>Cu≤150.00 mg/kg	0.124mg/kg<br>符合規定 Conform
- 砷 Arsenic (As)	不得過 41.67mg/kg 或 1500μg/日<br>As≤41.67mg/kg or 1,500μg/day	0.234mg/kg（8.42μg/日 day）<br>符合規定 Conform
- 鎘 Cadmium (Cd)	不得過 97.22mg/kg 或 3500μg/劑<br>Cd≤97.22mg/kg or 3,500μg/dose	0.008mg/kg（0.29μg/劑 dose）<br>符合規定 Conform
- 鉛 Lead (Pb)	不得過 4.97mg/kg 或 179μg/日<br>Pb≤4.97mg/kg or 179μg/day	0.063mg/kg（2.27μg/日 day）<br>符合規定 Conform
- 汞 Mercury (Hg)	不得過 1.00mg/kg 或 36μg/日<br>Hg≤1.00mg/kg or 36μg/day	＜0.001mg/kg（＜0.04μg/日 day）<br>符合規定 Conform
7. 農藥殘留# Pesticides Residues		
- 艾氏劑及狄氏劑 Aldrin & Dieldrin	艾氏劑及狄氏劑兩者之和≤0.05 毫克/千克<br>Sum of aldrin & dieldrin≤0.05 mg/kg<br>（檢出限艾氏劑 1.77μg/kg,狄氏劑 2.43μg/kg<br>LOD aldrin 1.77μg/kg, dieldrin 2.43μg/kg）	未檢出 Not detected<br>符合規定 Conform
- 氯丹 Chlordane	順式-,反式及氧化氯丹之和≤0.05 毫克/千克<br>Sum of cis-,trans- & oxy-chlordane≤0.05 mg/kg<br>（檢出限順式氯丹 2.05μg/kg, 反式氯丹 1.81μg/kg, 氧化氯丹 2.21μg/kg<br>LOD cis- chlordane 2.05μg/kg, trans- chlordane 1.81μg/kg, oxy-chlordane 2.21μg/kg）	未檢出 Not detected<br>符合規定 Conform
- 滴滴涕 DDT	4,4'-滴滴涕, 2,4'-滴滴涕, 4,4'-滴滴伊及 4,4'-滴滴滴之和≤1.0 毫克/千克<br>Sum of *p, p'*-DDT, *o,p'*-DDT, *p,p'*-DDE, *p,p'*-TDE ≤1.0 mg/kg<br>（檢出限 4,4'-滴滴涕 3.73μg/kg, 2,4'-滴滴涕 4.48μg/kg, 4,4'-滴滴伊 2.29μg/kg, 4,4'-滴滴滴 2.88μg/kg<br>LOD *p, p'*-DDT 3.73μg/kg, *o,p'*-DDT 4.48μg/kg, *p,p'*-DDE 2.29μg/kg, *p,p'*-TDE 2.88μg/kg）	未檢出 Not detected<br>符合規定 Conform
- 異狄氏劑 Endrin	≤0.05 mg/kg<br>（檢出限 LOD 3.78μg/kg）	未檢出 Not detected<br>符合規定 Conform

****

編制 Report compiled by：[signature]　　審核 Reviewed by：[signature]

培力（南寧）藥業有限公司檢測中心 PuraPharm (Nanning) Pharmaceuticals Co.,Ltd. Testing Laboratory
郵編(Postal No.)：530007　電話(Tel)：0771-3218026　傳真(Fax)：0771-3216602

- 七氯 Heptachlor	七氯及環氧七氯之和≤0.05 毫克/千克<br>Sum of heptachlor & heptachlor epoxide≤0.05 mg/kg<br>（檢出限七氯 1.78μg/kg, 環氧七氯 1.97μg/kg<br>LOD heptachlor 1.78μg/kg, heptachlor epoxide 1.97μg/kg）	未檢出 Not detected<br>符合規定 Conform
- 六氯苯 Hexachlorobenzene	≤0.1 mg/kg<br>（檢出限 LOD 2.16μg/kg）	未檢出 Not detected<br>符合規定 Conform
- 六六六 Hexachlorocyclohexane	α-,β-及 δ-異構體之和≤0.3 毫克/千克<br>Sum of α-,β-,δ- isomers≤0.3 mg/kg<br>（檢出限 α-六六六 1.61μg/kg, β-六六六 2.96μg/kg, δ-六六六 1.43μg/kg<br>LOD α- HCH 1.61μg/kg, β- HCH 2.96μg/kg, δ- HCH 1.43μg/kg）	未檢出 Not detected<br>符合規定 Conform
- 林丹 Lindane	≤0.6 mg/kg<br>（檢出限 LOD 1.56μg/kg）	未檢出 Not detected<br>符合規定 Conform
- 五氯硝基苯 Quintozene	五氯硝基苯，五氯苯胺及甲基五氯硫基苯之和≤1.0 毫克/千克<br>Sum of quintozene, pentachloroaniline and methyl pentachlorophenyl sulphide≤1.0 mg/kg<br>（檢出限五氯硝基苯 1.92μg/kg, 五氯苯胺 1.59μg/kg, 甲基五氯硫基苯 1.33μg/kg<br>LOD quintozene 1.92μg/kg , pentachloroaniline 1.59μg/kg , methyl pentachlorophenyl sulphide 1.33μg/kg）	未檢出 Not detected<br>符合規定 Conform
8. 微生物限度#<br>Microbial Limit		
- 需氧菌總數 TAMC (Total Aerobic Microbial Count)	≤500cfu/g	＜10cfu/g<br>符合規定 Conform
-霉菌和酵母菌總數 TYMC (Total Yeast and Mold Microbial Count)	≤100cfu/g	＜10cfu/g<br>符合規定 Conform
- 大腸埃希菌 Escherichia Coli	不得檢出/克 Not detected/g	未檢出/克 Not detected/g<br>符合規定 Conform

****

編制 Report compiled by：[signature] 審核 Reviewed by：[signature]

培力（南寧）藥業有限公司檢測中心 PuraPharm (Nanning) Pharmaceuticals Co.,Ltd. Testing Laboratory
郵編(Postal No.)：530007 電話(Tel)：0771-3218026 傳真(Fax)：0771-3216602

註：Remarks

1、 報告無“檢驗/檢測報告專用章”和缺騎縫章無效。

Test report without the stamp of "For Test/Test Report Only" is not authorized.

2、 複製的報告未重新加蓋“檢驗/檢測報告專用章”及騎縫章無效。

Certified copy of test reportwithout the stamp of"For Test/Test Report Only" is not authorized.

3、 報告無編制、審核及本中心代表簽字無效。

Test report must be authorized by the signature of the bodies of preparation, verification and representative of the testing centre.

4、 報告塗改、缺頁無效。

Test report with modification or lacking of page(s) is not authorized.

5、 對報告若有異議，請於收到報告之日起十五日內向檢驗單位提出書面申訴，否則按認可檢驗報告處理。本中心異議受理電話 0771-3218026。

The client is aggrieved by the test result. A request for review shall state in writing the reasons relied upon and shall be made to the testing centre within 15 days after receipt of the authorized test report. No requirement will be accepted after the definite time. Inquiry hot line is 0771-3218026.

6、 送樣委託檢驗，樣品名稱為委託單位自報名稱，報告僅對來樣負責。部份複製檢驗/檢測報告無效。

Name of tested product is provided by the client. The report will refer only to the sample tested. Copy of partial of the test report is not authorized.

# - 其他微生物限度要求: Other requirements for Microbial Limit Test

1. 含動物類藥材（包括提取物）的中藥固體製劑，每 10g 不得檢出沙門菌。

TCM solid preparation containing animal raw materials (including extract): Absence of Salmonella (10g).

2. 含藥材原粉的中藥固體製劑，每 10g 不得檢出沙門菌；耐膽鹽革蘭陰性菌應小於 10^2cfu（1g）。

TCM solid preparation containing herbal raw powders: Absence of Salmonella (10g); Not more than 10^2cfu of Bile-tolerant gram-negative bacteria (1g).

*** End of Report 報告完 ***

培力（南寧）藥業有限公司檢測中心 PuraPharm (Nanning) Pharmaceuticals Co.,Ltd. Testing Laboratory

郵編(Postal No.)：530007　　電話(Tel)：0771-3218026　　傳真(Fax)：0771-3216602

培力（南寧）藥業有限公司檢測中心 **PuraPharm (Nanning) Pharmaceuticals Co.,Ltd. Testing Laboratory**
中國廣西南寧市高新技術開發區 科園大道 46 號
No.46, Ke Yuan Road,Nanning New & High-tech Industrial Development Zone, Guangxi,China.

對“當歸配方顆粒”之樣品之分析報告
Report on the submitted sample identified by the client - Dang Gui

樣品名稱 Product Description	當歸配方顆粒 Dang Gui
樣品編號 Product Code	1017
樣品規格 Product Specification	200 克/瓶 g/ bottle
批號 Batch No.	A1600513
有效期至(年/月/日)Expiry Date（yyyy/mm/dd）	2019/04/23
本批數量 Quantity Produced	18828 瓶 bottles
樣品收到時狀態 Sample Receiving Condition	室溫下存放於密封塑膠樽原來包裝中<br>In sealed bottle of the original package under ambient condition.
製造商 Manufacturer	生產部 Production Department
委託檢驗單位 Inspected Entity	生產部 Production Department
來源地 Region of Origin	生產部 Production Department
目的地 Region of Destination	培力（南寧）藥業有限公司檢測中心<br>PuraPharm (Nanning) Pharmaceuticals Co.,Ltd. Testing Laboratory
檢驗日期(年/月/日)Testing Period（yyyy/mm/dd）	2016/05/04 - 2016/05/09

測試項目、分析方法及分析結果 Test Requested, Test Method and Test Results
請參考續頁。Please refer to the following page(s)

****

培力（南寧）藥業有限公司檢測中心代表簽名
Signed for and on behalf of PuraPharm (Nanning) Pharmaceuticals Co.,Ltd. Testing Laboratory

培力（南宁）药业有限公司检测中心 PURAPHARM (NANNING) PHARMACEUTICALS CO., TESTING LABORATORY 检测报告专用章 THE SPECIAL STAMP OF TEST REPORT 4501000241336

測試項目及分析方法 Test Requested and Test Method

測試項目 Test Items	參考方法 Reference Method
1. 性狀 Appearance	中國藥典, 2015 年版, 第四部, 通則 0104<br>The Pharmacopoeia of the People's Republic of China 2015, Vol.4, General chapter 0104
2. 鑒別 Identification	中國藥典, 2015 年版, 第四部, 通則 0502<br>The Pharmacopoeia of the People's Republic of China 2015, Vol.4, General chapter 0502
3. 浸出物 Extractives	中國藥典, 2015 年版, 第四部, 通則 2201<br>The Pharmacopoeia of the People's Republic of China 2015, Vol.4, General chapter 2201
4. 水分 Determination of Water	中國藥典, 2015 年版, 第四部, 通則 0832<br>The Pharmacopoeia of the People's Republic of China 2015, Vol.4, General chapter 0832
5. 粒度 Size of Granule	中國藥典, 2015 年版, 第四部, 通則 0982<br>The Pharmacopoeia of the People's Republic of China 2015, Vol.4, General chapter 0982
6. 溶化性<br>Determination of Dispersibility	中國藥典, 2015 年版, 第四部, 通則 0104<br>The Pharmacopoeia of the People's Republic of China 2015, Vol.4, General chapter 0104
7. 裝量 Capacity	中國藥典, 2015 年版, 第四部, 通則 0942<br>The Pharmacopoeia of the People's Republic of China 2015, Vol.4, General chapter 0942
8. 重金屬及有害元素<br>Heavy Metals and Toxic Elements	中國藥典, 2015 年版, 第四部, 通則 2321；電感耦合等離子體質譜法；<br>The Pharmacopoeia of the People's Republic of China 2015, Vol.4, General chapter 2321；ICP-MS
9. 農藥殘留 Pesticides Residues	香港中醫藥管理委員會《中成藥註冊申請手冊》<br>Chinese Medicine Council of Hong Kong《Application Form: Registration of proprietary Chinese medicines》
10. 微生物限度 Microbial Limit	中國藥典, 2015 年版, 第四部, 通則 1105、1106、1107<br>The Pharmacopoeia of the People's Republic of China 2015, Vol.4, General chapter 1105，1106，1107
- 需氧菌總數 TAMC (Total Aerobic Microbial Count)	
- 霉菌和酵母菌總數 TYMC (Total Yeast and Mold Microbial Count)	
- 大腸埃希菌 Escherichia Coli	
11. 含量測定 Assay	中國藥典, 2015 年版, 第四部, 通則 0512<br>The Pharmacopoeia of the People's Republic of China 2015, Vol.4, General chapter 0512

****

編制 Report compiled by：　　審核 Reviewed by：

培力（南寧）藥業有限公司檢測中心 PuraPharm (Nanning) Pharmaceuticals Co.,Ltd. Testing Laboratory
郵編(Postal No.)：530007　電話(Tel)：0771-3218026　傳真(Fax)：0771-3216602

分析結果 Test Results

測試項目 Test Items	品質標準 Quality Specification	當歸配方顆粒 Dang Gui
1. 性狀 Appearance	本品為黃白色至黃棕色顆粒，有香氣，味甘、辛、微苦。The product is yellowish white to yellowish brown granule, aromatic odour, sweet with pungent and slightly bitter taste.	符合規定 Conform
2. 鑒別 Identification	應符合規定 Shall meet the requirement	符合規定 Conform
3. 浸出物 Extractives	不得少於 15.0% No less than 15.0%	23.6%<br>符合規定 Conform
4. 水分 Determination of Water	≤6.5% (W/W)	4.4%<br>符合規定 Conform
5. 粒度 Size of Granule	不能通過一號篩和能通過五號篩的顆粒和粉末總和不得超過 15% Sum of weight of granules that cannot pass through sieve No.1 and weight of powder that can pass through sieve No.5≤15%	8%<br>符合規定 Conform
6. 溶化性<br>Determination of Dispersibility	應符合規定 Shall meet the requirement	符合規定 Conform
7. 裝量 Capacity	應符合規定 Shall meet the requirement	符合規定 Conform
8. 重金屬及有害元素#<br>Heavy Metals and Toxic Elements		
- 銅 Copper (Cu)	不得過 150.00mg/kg<br>Cu≤150.00 mg/kg	0.808mg/kg<br>符合規定 Conform
- 砷 Arsenic (As)	不得過 41.67mg/kg 或 1500μg/日<br>As≤41.67mg/kg or 1,500μg/day	0.129mg/kg（4.64μg/日 day）<br>符合規定 Conform
- 鎘 Cadmium (Cd)	不得過 97.22mg/kg 或 3500μg/劑<br>Cd≤97.22mg/kg or 3,500μg/dose	0.002mg/kg（0.07μg/劑 dose）<br>符合規定 Conform
- 鉛 Lead (Pb)	不得過 4.97mg/kg 或 179μg/日<br>Pb≤4.97mg/kg or 179μg/day	0.050mg/kg（1.80μg/日 day）<br>符合規定 Conform
- 汞 Mercury (Hg)	不得過 1.00mg/kg 或 36μg/日<br>Hg≤1.00mg/kg or 36μg/day	＜0.001mg/kg（＜0.04μg/日 day）<br>符合規定 Conform
9. 農藥殘留#<br>Pesticides Residues		
- 艾氏劑及狄氏劑<br>Aldrin & Dieldrin	艾氏劑及狄氏劑兩者之和≤0.05 毫克/千克<br>Sum of aldrin & dieldrin≤0.05 mg/kg<br>（檢出限艾氏劑 1.77μg/kg,狄氏劑 2.43μg/kg<br>LOD aldrin 1.77μg/kg, dieldrin 2.43μg/kg）	未檢出 Not detected<br>符合規定 Conform
- 氯丹 Chlordane	順式-,反式及氧化氯丹之和≤0.05 毫克/千克<br>Sum of cis-,trans- & oxy-chlordane≤0.05 mg/kg<br>（檢出限順式氯丹 2.05μg/kg, 反式氯丹 1.81μg/kg, 氧化氯丹 2.21μg/kg<br>LOD cis- chlordane 2.05μg/kg, trans- chlordane 1.81μg/kg, oxy-chlordane 2.21μg/kg）	未檢出 Not detected<br>符合規定 Conform
- 滴滴涕 DDT	4,4'-滴滴涕, 2,4'-滴滴涕, 4,4'-滴滴伊及 4,4'-滴滴滴之和≤1.0 毫克/千克<br>Sum of *p, p*'-DDT, *o,p*'-DDT, *p,p*'-DDE, *p,p*'-TDE ≤1.0 mg/kg<br>（檢出限 4,4'-滴滴涕 3.73μg/kg, 2,4'-滴滴涕 4.48μg/kg, 4,4'-滴滴伊 2.29μg/kg, 4,4'-滴滴滴 2.88μg/kg<br>LOD *p, p*'-DDT 3.73μg/kg, *o,p*'-DDT 4.48μg/kg, *p,p*'-DDE 2.29μg/kg, *p,p*'-TDE 2.88μg/kg）	未檢出 Not detected<br>符合規定 Conform
- 異狄氏劑 Endrin	≤0.05 mg/kg<br>（檢出限 LOD 3.78μg/kg）	未檢出 Not detected<br>符合規定 Conform

****

編制 Report compiled by：[signature] 審核 Reviewed by：[signature]

- 七氯 Heptachlor	七氯及環氧七氯之和≤0.05 毫克/千克<br>Sum of heptachlor & heptachlor epoxide≤0.05 mg/kg<br>（檢出限七氯 1.78μg/kg, 環氧七氯 1.97μg/kg<br>LOD heptachlor 1.78μg/kg, heptachlor epoxide 1.97μg/kg）	未檢出 Not detected<br>符合規定 Conform
- 六氯苯 Hexachlorobenzene	≤0.1 mg/kg<br>（檢出限 LOD 2.16μg/kg）	未檢出 Not detected<br>符合規定 Conform
- 六六六 Hexachlorocyclohexane	α-,β-及 δ-異構體之和≤0.3 毫克/千克<br>Sum of α-,β-,δ- isomers≤0.3 mg/kg<br>（檢出限 α-六六六 1.61μg/kg, β-六六六 2.96μg/kg, δ-六六六 1.43μg/kg<br>LOD α- HCH 1.61μg/kg, β- HCH 2.96μg/kg, δ- HCH 1.43μg/kg）	未檢出 Not detected<br>符合規定 Conform
- 林丹 Lindane	≤0.6 mg/kg<br>（檢出限 LOD 1.56μg/kg）	未檢出 Not detected<br>符合規定 Conform
- 五氯硝基苯 Quintozene	五氯硝基苯，五氯苯胺及甲基五氯硫基苯之和≤1.0 毫克/千克<br>Sum of quintozene, pentachloroaniline and methyl pentachlorophenyl sulphide≤1.0 mg/kg<br>（檢出限五氯硝基苯 1.92μg/kg, 五氯苯胺 1.59μg/kg, 甲基五氯硫基苯 1.33μg/kg<br>LOD quintozene 1.92μg/kg , pentachloroaniline 1.59μg/kg , methyl pentachlorophenyl sulphide 1.33μg/kg）	未檢出 Not detected<br>符合規定 Conform
10. 微生物限度#<br>Microbial Limit		
- 需氧菌總數 TAMC (Total Aerobic Microbial Count)	≤500cfu/g	15cfu/g<br>符合規定 Conform
-霉菌和酵母菌總數 TYMC (Total Yeast and Mold Microbial Count)	≤100cfu/g	＜10cfu/g<br>符合規定 Conform
- 大腸埃希菌 Escherichia Coli	不得檢出/克 Not detected/g	未檢出/克 Not detected/g<br>符合規定 Conform
11. 含量測定 Assay	含阿魏酸不得少於 0.15mg/g<br>Ferulic acid no less than 0.15mg/g	0.47mg/g<br>符合規定 Conform

****

編制 Report compiled by：[signature]　　審核 Reviewed by：[signature]

培力（南寧）藥業有限公司檢測中心 PuraPharm (Nanning) Pharmaceuticals Co.,Ltd. Testing Laboratory
郵編(Postal No.)：530007　電話(Tel)：0771-3218026　傳真(Fax)：0771-3216602

註：Remarks

1、 報告無“檢驗/檢測報告專用章”和缺騎縫章無效。

Test report without the stamp of "For Test/Test Report Only" is not authorized.

2、 複製的報告未重新加蓋“檢驗/檢測報告專用章”及騎縫章無效。

Certified copy of test reportwithout the stamp of"For Test/Test Report Only" is not authorized.

3、 報告無編制、審核及本中心代表簽字無效。

Test report must be authorized by the signature of the bodies of preparation, verification and representative of the testing centre.

4、 報告塗改、缺頁無效。

Test report with modification or lacking of page(s) is not authorized.

5、 對報告若有異議，請於收到報告之日起十五日內向檢驗單位提出書面申訴，否則按認可檢驗報告處理。本中心異議受理電話 0771-3218026。

The client is aggrieved by the test result. A request for review shall state in writing the reasons relied upon and shall be made to the testing centre within 15 days after receipt of the authorized test report. No requirement will be accepted after the definite time. Inquiry hot line is 0771-3218026.

6、 送樣委託檢驗，樣品名稱為委託單位自報名稱，報告僅對來樣負責。部份複製檢驗/檢測報告無效。

Name of tested product is provided by the client. The report will refer only to the sample tested. Copy of partial of the test report is not authorized.

# - 其他微生物限度要求: Other requirements for Microbial Limit Test

1. 含動物類藥材（包括提取物）的中藥固體製劑，每 10g 不得檢出沙門菌。

TCM solid preparation containing animal raw materials (including extract): Absence of Salmonella (10g).

2. 含藥材原粉的中藥固體製劑，每 10g 不得檢出沙門菌；耐膽鹽革蘭陰性菌應小於 10^2cfu（1g）。

TCM solid preparation containing herbal raw powders: Absence of Salmonella (10g); Not more than 10^2cfu of Bile-tolerant gram-negative bacteria (1g).

*** End of Report 報告完 ***

檢驗報告 Test Report

培力（南寧）藥業有限公司檢測中心 PuraPharm (Nanning) Pharmaceuticals Co.,Ltd. Testing Laboratory
中國廣西南寧市高新技術開發區 科園大道 46 號
No.46, Ke Yuan Road,Nanning New & High-tech Industrial Development Zone, Guangxi,China.

對 "地黃配方顆粒" 之樣品之分析報告

Report on the submitted sample identified by the client - Di Huang

樣品名稱 Product Description	地黃配方顆粒 Di Huang
樣品編號 Product Code	1030
樣品規格 Product Specification	200 克/瓶 g/ bottle
批號 Batch No.	A1600007
有效期至(年/月/日)Expiry Date（yyyy/mm/dd）	2019/04/25
本批數量 Quantity Produced	14742 瓶 bottles
樣品收到時狀態 Sample Receiving Condition	室溫下存放於密封塑膠樽原來包裝中<br>In sealed bottle of the original package under ambient condition.
製造商 Manufacturer	生產部 Production Department
委託檢驗單位 Inspected Entity	生產部 Production Department
來源地 Region of Origin	生產部 Production Department
目的地 Region of Destination	培力（南寧）藥業有限公司檢測中心<br>PuraPharm (Nanning) Pharmaceuticals Co.,Ltd. Testing Laboratory
檢驗日期(年/月/日)Testing Period（yyyy/mm/dd）	2016/04/29 - 2016/05/04

測試項目、分析方法及分析結果 Test Requested, Test Method and Test Results

請參考續頁。Please refer to the following page(s)

****

培力（南寧）藥業有限公司檢測中心代表簽名

Signed for and on behalf of PuraPharm (Nanning) Pharmaceuticals Co.,Ltd. Testing Laboratory

測試項目及分析方法 Test Requested and Test Method

測試項目 Test Items	參考方法 Reference Method
1. 性狀 Appearance	中國藥典, 2015 年版, 第四部, 通則 0104<br>The Pharmacopoeia of the People's Republic of China 2015, Vol.4, General chapter 0104
2. 水分 Determination of Water	中國藥典, 2015 年版, 第四部, 通則 0832<br>The Pharmacopoeia of the People's Republic of China 2015, Vol.4, General chapter 0832
3. 粒度 Size of Granule	中國藥典, 2015 年版, 第四部, 通則 0982<br>The Pharmacopoeia of the People's Republic of China 2015, Vol.4, General chapter 0982
4. 溶化性<br>Determination of Dispersibility	中國藥典, 2015 年版, 第四部, 通則 0104<br>The Pharmacopoeia of the People's Republic of China 2015, Vol.4, General chapter 0104
5. 裝量 Capacity	中國藥典, 2015 年版, 第四部, 通則 0942<br>The Pharmacopoeia of the People's Republic of China 2015, Vol.4, General chapter 0942
6. 重金屬及有害元素<br>Heavy Metals and Toxic Elements	中國藥典, 2015 年版, 第四部, 通則 2321；電感耦合等離子體質譜法；<br>The Pharmacopoeia of the People's Republic of China 2015, Vol.4, General chapter 2321；ICP-MS
7. 農藥殘留 Pesticides Residues	香港中醫藥管理委員會《中成藥註冊申請手冊》<br>Chinese Medicine Council of Hong Kong《Application Form: Registration of proprietary Chinese medicines》
8. 微生物限度 Microbial Limit	中國藥典, 2015 年版, 第四部, 通則 1105、1106、1107<br>The Pharmacopoeia of the People's Republic of China 2015, Vol.4, General chapter 1105，1106，1107
- 需氧菌總數 TAMC (Total Aerobic Microbial Count)	
- 霉菌和酵母菌總數 TYMC (Total Yeast and Mold Microbial Count)	
- 大腸埃希菌 Escherichia Coli	

****

編制 Report compiled by：罗昌彬　　審核 Reviewed by：杨冬苗

分析結果 Test Results

測試項目 Test Items	品質標準 Quality Specification	地黃配方顆粒 Di Huang
1. 性狀 Appearance	本品為淡灰黃色至棕褐色顆粒，氣微，味微甜、微苦。The prodcut is light greyish yellow to brown granule, slight odour, slightly sweet and bitter taste.	符合規定 Conform
2. 水分 Determination of Water	≤6.5% (W/W)	3.6%<br>符合規定 Conform
3. 粒度 Size of Granule	不能通過一號篩和能通過五號篩的顆粒和粉末總和不得超過 15% Sum of weight of granules that cannot pass through sieve No.1 and weight of powder that can pass through sieve No.5≤15%	5%<br>符合規定 Conform
4. 溶化性<br>Determination of Dispersibility	應符合規定 Shall meet the requirement	符合規定 Conform
5. 裝量 Capacity	應符合規定 Shall meet the requirement	符合規定 Conform
6. 重金屬及有害元素#<br>Heavy Metals and Toxic Elements		
- 銅 Copper (Cu)	不得過 150.00mg/kg<br>Cu≤150.00 mg/kg	0.189mg/kg<br>符合規定 Conform
- 砷 Arsenic (As)	不得過 41.67mg/kg 或 1500μg/日<br>As≤41.67mg/kg or 1,500μg/day	0.284mg/kg（10.22μg/日 day）<br>符合規定 Conform
- 鎘 Cadmium (Cd)	不得過 97.22mg/kg 或 3500μg/劑<br>Cd≤97.22mg/kg or 3,500μg/dose	0.003mg/kg（0.11μg/劑 dose）<br>符合規定 Conform
- 鉛 Lead (Pb)	不得過 4.97mg/kg 或 179μg/日<br>Pb≤4.97mg/kg or 179μg/day	0.079mg/kg（2.84μg/日 day）<br>符合規定 Conform
- 汞 Mercury (Hg)	不得過 1.00mg/kg 或 36μg/日<br>Hg≤1.00mg/kg or 36μg/day	＜0.001mg/kg（＜0.04μg/日 day）<br>符合規定 Conform
7. 農藥殘留#<br>Pesticides Residues		
- 艾氏劑及狄氏劑<br>Aldrin & Dieldrin	艾氏劑及狄氏劑兩者之和≤0.05 毫克/千克<br>Sum of aldrin & dieldrin≤0.05 mg/kg<br>（檢出限艾氏劑 1.77μg/kg,狄氏劑 2.43μg/kg<br>LOD aldrin 1.77μg/kg, dieldrin 2.43μg/kg）	未檢出 Not detected<br>符合規定 Conform
- 氯丹 Chlordane	順式-,反式及氧化氯丹之和≤0.05 毫克/千克<br>Sum of cis-,trans- & oxy-chlordane≤0.05 mg/kg<br>（檢出限順式氯丹 2.05μg/kg, 反式氯丹 1.81μg/kg, 氧化氯丹 2.21μg/kg<br>LOD cis- chlordane 2.05μg/kg, trans- chlordane 1.81μg/kg, oxy-chlordane 2.21μg/kg）	未檢出 Not detected<br>符合規定 Conform
- 滴滴涕 DDT	4,4`-滴滴涕, 2,4`-滴滴涕, 4,4`-滴滴伊及 4,4`-滴滴滴之和≤1.0 毫克/千克<br>Sum of *p, p*`-DDT, *o,p*`-DDT, *p,p*`-DDE, *p,p*`-TDE ≤1.0 mg/kg<br>（檢出限 4,4`-滴滴涕 3.73μg/kg, 2,4`-滴滴涕 4.48μg/kg, 4,4`-滴滴伊 2.29μg/kg, 4,4`-滴滴滴 2.88μg/kg<br>LOD *p, p*`-DDT 3.73μg/kg, *o,p*`-DDT 4.48μg/kg, *p,p*`-DDE 2.29μg/kg, *p,p*`-TDE 2.88μg/kg）	未檢出 Not detected<br>符合規定 Conform
- 異狄氏劑 Endrin	≤0.05 mg/kg<br>（檢出限 LOD 3.78μg/kg）	未檢出 Not detected<br>符合規定 Conform

****

編制 Report compiled by：[signature] 審核 Reviewed by：[signature]

培力（南寧）藥業有限公司檢測中心 PuraPharm (Nanning) Pharmaceuticals Co.,Ltd. Testing Laboratory
郵編(Postal No.)：530007 電話(Tel)：0771-3218026 傳真(Fax)：0771-3216602

- 七氯 Heptachlor	七氯及環氧七氯之和≤0.05 毫克/千克<br>Sum of heptachlor & heptachlor epoxide≤0.05 mg/kg<br>（檢出限七氯 1.78μg/kg，環氧七氯 1.97μg/kg<br>LOD heptachlor 1.78μg/kg, heptachlor epoxide 1.97μg/kg）	未檢出 Not detected<br>符合規定 Conform
- 六氯苯 Hexachlorobenzene	≤0.1 mg/kg<br>（檢出限 LOD 2.16μg/kg）	未檢出 Not detected<br>符合規定 Conform
- 六六六 Hexachlorocyclohexane	α-,β-及 δ-異構體之和≤0.3 毫克/千克<br>Sum of α-,β-,δ- isomers≤0.3 mg/kg<br>（檢出限 α-六六六 1.61μg/kg, β-六六六 2.96μg/kg, δ-六六六 1.43μg/kg<br>LOD α- HCH 1.61μg/kg, β- HCH 2.96μg/kg, δ- HCH 1.43μg/kg）	4.76×10^{-3}mg/kg<br>符合規定 Conform
- 林丹 Lindane	≤0.6 mg/kg<br>（檢出限 LOD 1.56μg/kg）	未檢出 Not detected<br>符合規定 Conform
- 五氯硝基苯 Quintozene	五氯硝基苯，五氯苯胺及甲基五氯硫基苯之和≤1.0 毫克/千克<br>Sum of quintozene, pentachloroaniline and methyl pentachlorophenyl sulphide≤1.0 mg/kg<br>（檢出限五氯硝基苯 1.92μg/kg，五氯苯胺 1.59μg/kg，甲基五氯硫基苯 1.33μg/kg<br>LOD quintozene 1.92μg/kg , pentachloroaniline 1.59μg/kg , methyl pentachlorophenyl sulphide 1.33μg/kg）	未檢出 Not detected<br>符合規定 Conform
8. 微生物限度#<br>Microbial Limit		
- 需氧菌總數 TAMC (Total Aerobic Microbial Count)	≤500cfu/g	45cfu/g<br>符合規定 Conform
-霉菌和酵母菌總數 TYMC (Total Yeast and Mold Microbial Count)	≤100cfu/g	＜10cfu/g<br>符合規定 Conform
- 大腸埃希菌 Escherichia Coli	不得檢出/克 Not detected/g	未檢出/克 Not detected/g<br>符合規定 Conform

****

編制 Report compiled by：　　　審核 Reviewed by：

培力（南寧）藥業有限公司檢測中心 PuraPharm (Nanning) Pharmaceuticals Co.,Ltd. Testing Laboratory
郵編(Postal No.)：530007　電話(Tel)：0771-3218026　傳真(Fax)：0771-3216602

註：Remarks

1、 報告無"檢驗/檢測報告專用章" 和缺騎縫章無效。

Test report without the stamp of "For Test/Test Report Only" is not authorized.

2、 複製的報告未重新加蓋"檢驗/檢測報告專用章" 及騎縫章無效。

Certified copy of test reportwithout the stamp of"For Test/Test Report Only" is not authorized.

3、 報告無編制、審核及本中心代表簽字無效。

Test report must be authorized by the signature of the bodies of preparation, verification and representative of the testing centre.

4、 報告塗改、缺頁無效。

Test report with modification or lacking of page(s) is not authorized.

5、 對報告若有異議，請於收到報告之日起十五日內向檢驗單位提出書面申訴，否則按認可檢驗報告處理。本中心異議受理電話 0771-3218026。

The client is aggrieved by the test result. A request for review shall state in writing the reasons relied upon and shall be made to the testing centre within 15 days after receipt of the authorized test report. No requirement will be accepted after the definite time. Inquiry hot line is 0771-3218026.

6、 送樣委託檢驗，樣品名稱為委託單位自報名稱，報告僅對來樣負責。部份複製檢驗/檢測報告無效。

Name of tested product is provided by the client. The report will refer only to the sample tested. Copy of partial of the test report is not authorized.

# - 其他微生物限度要求: Other requirements for Microbial Limit Test

1. 含動物類藥材（包括提取物）的中藥固體製劑，每 10g 不得檢出沙門菌。

TCM solid preparation containing animal raw materials (including extract): Absence of Salmonella (10g).

2. 含藥材原粉的中藥固體製劑，每 10g 不得檢出沙門菌；耐膽鹽革蘭陰性菌應小於 10^2cfu（1g）。

TCM solid preparation containing herbal raw powders: Absence of Salmonella (10g); Not more than 10^2cfu of Bile-tolerant gram-negative bacteria (1g).

*** End of Report 報告完 ***

培力（南寧）藥業有限公司檢測中心 PuraPharm (Nanning) Pharmaceuticals Co.,Ltd. Testing Laboratory
郵編(Postal No.)：530007 電話(Tel)：0771-3218026 傳真(Fax)：0771-3216602

培力（南寧）藥業有限公司檢測中心 **PuraPharm (Nanning) Pharmaceuticals Co.,Ltd. Testing Laboratory**
中國廣西南寧市高新技術開發區 No.46, Ke Yuan Road,Nanning New & High-tech
科園大道 46 號 Industrial Development Zone, Guangxi,China.

對“麥冬配方顆粒”之樣品之分析報告

Report on the submitted sample identified by the client - Mai Dong

樣品名稱 Product Description	麥冬配方顆粒 Mai Dong
樣品編號 Product Code	1036
樣品規格 Product Specification	200 克/瓶 g/ bottle
批號 Batch No.	A1600447
有效期至(年/月/日)Expiry Date（yyyy/mm/dd）	2019/05/01
本批數量 Quantity Produced	17614 瓶 bottles
樣品收到時狀態 Sample Receiving Condition	室溫下存放於密封塑膠樽原來包裝中<br>In sealed bottle of the original package under ambient condition.
製造商 Manufacturer	生產部 Production Department
委託檢驗單位 Inspected Entity	生產部 Production Department
來源地 Region of Origin	生產部 Production Department
目的地 Region of Destination	培力（南寧）藥業有限公司檢測中心<br>PuraPharm (Nanning) Pharmaceuticals Co.,Ltd. Testing Laboratory
檢驗日期(年/月/日)Testing Period（yyyy/mm/dd）	2016/05/10 - 2016/05/16

測試項目、分析方法及分析結果 Test Requested, Test Method and Test Results

請參考續頁。Please refer to the following page(s)

****

培力（南寧）藥業有限公司檢測中心代表簽名

Signed for and on behalf of PuraPharm (Nanning) Pharmaceuticals Co.,Ltd. Testing Laboratory

測試項目及分析方法 Test Requested and Test Method

測試項目 Test Items	參考方法 Reference Method
1. 性狀 Appearance	中國藥典, 2015 年版, 第四部, 通則 0104<br>The Pharmacopoeia of the People's Republic of China 2015, Vol.4, General chapter 0104
2. 水分 Determination of Water	中國藥典, 2015 年版, 第四部, 通則 0832<br>The Pharmacopoeia of the People's Republic of China 2015, Vol.4, General chapter 0832
3. 粒度 Size of Granule	中國藥典, 2015 年版, 第四部, 通則 0982<br>The Pharmacopoeia of the People's Republic of China 2015, Vol.4, General chapter 0982
4. 溶化性<br>Determination of Dispersibility	中國藥典, 2015 年版, 第四部, 通則 0104<br>The Pharmacopoeia of the People's Republic of China 2015, Vol.4, General chapter 0104
5. 裝量 Capacity	中國藥典, 2015 年版, 第四部, 通則 0942<br>The Pharmacopoeia of the People's Republic of China 2015, Vol.4, General chapter 0942
6. 重金屬及有害元素<br>Heavy Metals and Toxic Elements	中國藥典, 2015 年版, 第四部, 通則 2321；電感耦合等離子體質譜法；<br>The Pharmacopoeia of the People's Republic of China 2015, Vol.4, General chapter 2321；ICP-MS
7. 農藥殘留 Pesticides Residues	香港中醫藥管理委員會《中成藥註冊申請手冊》<br>Chinese Medicine Council of Hong Kong《Application Form: Registration of proprietary Chinese medicines》
8. 微生物限度 Microbial Limit	中國藥典, 2015 年版, 第四部, 通則 1105、1106、1107<br>The Pharmacopoeia of the People's Republic of China 2015, Vol.4, General chapter 1105，1106，1107
- 需氧菌總數 TAMC (Total Aerobic Microbial Count)	
- 霉菌和酵母菌總數 TYMC (Total Yeast and Mold Microbial Count)	
- 大腸埃希菌 Escherichia Coli	

****

培力（南寧）藥業有限公司檢測中心 PuraPharm (Nanning) Pharmaceuticals Co.,Ltd. Testing Laboratory
郵編(Postal No.)：530007 電話(Tel)：0771-3218026 傳真(Fax)：0771-3216602

分析結果 Test Results

測試項目 Test Items	品質標準 Quality Specification	麥冬配方顆粒 Mai Dong
1. 性狀 Appearance	本品為黃白色至淺黃色顆粒，氣微香，味甘。<br>The product is yellowish white to light yellow granule , slightly aromatic odour, sweet taste.	符合規定 Conform
2. 水分 Determination of Water	≤6.5% (W/W)	3.8%<br>符合規定 Conform
3. 粒度 Size of Granule	不能通過一號篩和能通過五號篩的顆粒和粉末總和不得超過 15% Sum of weight of granules that cannot pass through sieve No.1 and weight of powder that can pass through sieve No.5≤15%	14%<br>符合規定 Conform
4. 溶化性<br>Determination of Dispersibility	應符合規定 Shall meet the requirement	符合規定 Conform
5. 裝量 Capacity	應符合規定 Shall meet the requirement	符合規定 Conform
6. 重金屬及有害元素#<br>Heavy Metals and Toxic Elements		
- 銅 Copper (Cu)	不得過 150.00mg/kg<br>Cu≤150.00 mg/kg	0.224mg/kg<br>符合規定 Conform
- 砷 Arsenic (As)	不得過 41.67mg/kg 或 1500μg/日<br>As≤41.67mg/kg or 1,500μg/day	0.049mg/kg（1.76μg/日 day）<br>符合規定 Conform
- 鎘 Cadmium (Cd)	不得過 97.22mg/kg 或 3500μg/劑<br>Cd≤97.22mg/kg or 3,500μg/dose	0.006mg/kg（0.22μg/劑 dose）<br>符合規定 Conform
- 鉛 Lead (Pb)	不得過 4.97mg/kg 或 179μg/日<br>Pb≤4.97mg/kg or 179μg/day	0.021mg/kg（0.76μg/日 day）<br>符合規定 Conform
- 汞 Mercury (Hg)	不得過 1.00mg/kg 或 36μg/日<br>Hg≤1.00mg/kg or 36μg/day	＜0.001mg/kg（＜0.04μg/日 day）<br>符合規定 Conform
7. 農藥殘留#<br>Pesticides Residues		
- 艾氏劑及狄氏劑<br>Aldrin & Dieldrin	艾氏劑及狄氏劑兩者之和≤0.05 毫克/千克<br>Sum of aldrin & dieldrin≤0.05 mg/kg<br>（檢出限艾氏劑 1.77μg/kg,狄氏劑 2.43μg/kg<br>LOD aldrin 1.77μg/kg, dieldrin 2.43μg/kg）	未檢出 Not detected<br>符合規定 Conform
- 氯丹 Chlordane	順式-,反式及氧化氯丹之和≤0.05 毫克/千克<br>Sum of cis-,trans- & oxy-chlordane≤0.05 mg/kg<br>（檢出限順式氯丹 2.05μg/kg, 反式氯丹 1.81μg/kg, 氧化氯丹 2.21μg/kg<br>LOD cis- chlordane 2.05μg/kg, trans- chlordane 1.81μg/kg, oxy-chlordane 2.21μg/kg）	未檢出 Not detected<br>符合規定 Conform
- 滴滴涕 DDT	4,4'-滴滴涕, 2,4'-滴滴涕, 4,4'-滴滴伊及 4,4'-滴滴滴之和≤1.0 毫克/千克<br>Sum of *p, p*'-DDT, *o,p*'-DDT, *p,p*'-DDE, *p,p*'-TDE ≤1.0 mg/kg<br>（檢出限 4,4'-滴滴涕 3.73μg/kg, 2,4'-滴滴涕 4.48μg/kg, 4,4'-滴滴伊 2.29μg/kg, 4,4'-滴滴滴 2.88μg/kg<br>LOD *p, p*'-DDT 3.73μg/kg, *o,p*'-DDT 4.48μg/kg, *p,p*'-DDE 2.29μg/kg, *p,p*'-TDE 2.88μg/kg）	未檢出 Not detected<br>符合規定 Conform
- 異狄氏劑 Endrin	≤0.05 mg/kg<br>（檢出限 LOD 3.78μg/kg）	未檢出 Not detected<br>符合規定 Conform

****

編制 Report compiled by：[signature] 審核 Reviewed by：[signature]

培力（南寧）藥業有限公司檢測中心 PuraPharm (Nanning) Pharmaceuticals Co.,Ltd. Testing Laboratory
郵編(Postal No.)：530007 電話(Tel)：0771-3218026 傳真(Fax)：0771-3216602

- 七氯 Heptachlor	七氯及環氧七氯之和≤0.05 毫克/千克<br>Sum of heptachlor & heptachlor epoxide≤0.05 mg/kg<br>（檢出限七氯 1.78μg/kg, 環氧七氯 1.97μg/kg<br>LOD heptachlor 1.78μg/kg, heptachlor epoxide 1.97μg/kg）	未檢出 Not detected<br>符合規定 Conform
- 六氯苯 Hexachlorobenzene	≤0.1 mg/kg<br>（檢出限 LOD 2.16μg/kg）	未檢出 Not detected<br>符合規定 Conform
- 六六六 Hexachlorocyclohexane	α-,β-及 δ-異構體之和≤0.3 毫克/千克<br>Sum of α-,β-,δ- isomers≤0.3 mg/kg<br>（檢出限 α-六六六 1.61μg/kg, β-六六六 2.96μg/kg, δ-六六六 1.43μg/kg<br>LOD α- HCH 1.61μg/kg, β- HCH 2.96μg/kg, δ- HCH 1.43μg/kg）	未檢出 Not detected<br>符合規定 Conform
- 林丹 Lindane	≤0.6 mg/kg<br>（檢出限 LOD 1.56μg/kg）	未檢出 Not detected<br>符合規定 Conform
- 五氯硝基苯 Quintozene	五氯硝基苯，五氯苯胺及甲基五氯硫基苯之和≤1.0 毫克/千克<br>Sum of quintozene, pentachloroaniline and methyl pentachlorophenyl sulphide≤1.0 mg/kg<br>（檢出限五氯硝基苯 1.92μg/kg, 五氯苯胺 1.59μg/kg, 甲基五氯硫基苯 1.33μg/kg<br>LOD quintozene 1.92μg/kg , pentachloroaniline 1.59μg/kg , methyl pentachlorophenyl sulphide 1.33μg/kg）	未檢出 Not detected<br>符合規定 Conform
8. 微生物限度#<br>Microbial Limit		
-需氧菌總數 TAMC (Total Aerobic Microbial Count)	≤500cfu/g	＜10cfu/g<br>符合規定 Conform
-霉菌和酵母菌總數 TYMC (Total Yeast and Mold Microbial Count)	≤100cfu/g	＜10cfu/g<br>符合規定 Conform
- 大腸埃希菌 Escherichia Coli	不得檢出/克 Not detected/g	未檢出/克 Not detected/g<br>符合規定 Conform

****

編制 Report compiled by：[signature] 審核 Reviewed by：[signature]

培力（南寧）藥業有限公司檢測中心 PuraPharm (Nanning) Pharmaceuticals Co.,Ltd. Testing Laboratory
郵編(Postal No.)：530007 電話(Tel)：0771-3218026 傳真(Fax)：0771-3216602

註：Remarks

1、 報告無“檢驗/檢測報告專用章”和缺騎縫章無效。

Test report without the stamp of "For Test/Test Report Only" is not authorized.

2、 複製的報告未重新加蓋“檢驗/檢測報告專用章”及騎縫章無效。

Certified copy of test reportwithout the stamp of"For Test/Test Report Only" is not authorized.

3、 報告無編制、審核及本中心代表簽字無效。

Test report must be authorized by the signature of the bodies of preparation, verification and representative of the testing centre.

4、 報告塗改、缺頁無效。

Test report with modification or lacking of page(s) is not authorized.

5、 對報告若有異議，請於收到報告之日起十五日內向檢驗單位提出書面申訴，否則按認可檢驗報告處理。本中心異議受理電話 0771-3218026。

The client is aggrieved by the test result. A request for review shall state in writing the reasons relied upon and shall be made to the testing centre within 15 days after receipt of the authorized test report. No requirement will be accepted after the definite time. Inquiry hot line is 0771-3218026.

6、 送樣委託檢驗，樣品名稱為委託單位自報名稱，報告僅對來樣負責。部份複製檢驗/檢測報告無效。

Name of tested product is provided by the client. The report will refer only to the sample tested. Copy of partial of the test report is not authorized.

# - 其他微生物限度要求: Other requirements for Microbial Limit Test

1. 含動物類藥材（包括提取物）的中藥固體製劑，每 10g 不得檢出沙門菌。

TCM solid preparation containing animal raw materials (including extract): Absence of Salmonella (10g).

2. 含藥材原粉的中藥固體製劑，每 10g 不得檢出沙門菌；耐膽鹽革蘭陰性菌應小於 10^2cfu（1g）。

TCM solid preparation containing herbal raw powders: Absence of Salmonella (10g); Not more than 10^2cfu of Bile-tolerant gram-negative bacteria (1g).

*** End of Report 報告完 ***

培力（南寧）藥業有限公司檢測中心 **PuraPharm (Nanning) Pharmaceuticals Co.,Ltd. Testing Laboratory**

中國廣西南寧市高新技術開發區科園大道 46 號 No.46, Ke Yuan Road,Nanning New & High-tech Industrial Development Zone, Guangxi,China.

對"肉蓯蓉配方顆粒"之樣品之分析報告

Report on the submitted sample identified by the client - Rou Cong Rong

樣品名稱 Product Description	肉蓯蓉配方顆粒 Rou Cong Rong
樣品編號 Product Code	1060
樣品規格 Product Specification	200 克/瓶 g/ bottle
批號 Batch No.	A1600010
有效期至(年/月/日)Expiry Date（yyyy/mm/dd）	2019/02/27
本批數量 Quantity Produced	3007 瓶 bottles
樣品收到時狀態 Sample Receiving Condition	室溫下存放於密封塑膠樽原來包裝中<br>In sealed bottle of the original package under ambient condition.
製造商 Manufacturer	生產部 Production Department
委託檢驗單位 Inspected Entity	生產部 Production Department
來源地 Region of Origin	生產部 Production Department
目的地 Region of Destination	培力（南寧）藥業有限公司檢測中心<br>PuraPharm (Nanning) Pharmaceuticals Co.,Ltd. Testing Laboratory
檢驗日期(年/月/日)Testing Period（yyyy/mm/dd）	2016/03/02 - 2016/03/07

測試項目、分析方法及分析結果 Test Requested, Test Method and Test Results

請參考續頁。Please refer to the following page(s)

****

培力（南寧）藥業有限公司檢測中心代表簽名

Signed for and on behalf of PuraPharm (Nanning) Pharmaceuticals Co.,Ltd. Testing Laboratory

培力（南寧）藥業有限公司檢測中心 PuraPharm (Nanning) Pharmaceuticals Co.,Ltd. Testing Laboratory

郵編(Postal No.)：530007 電話(Tel)：0771-3218026 傳真(Fax)：0771-3216602

測試項目及分析方法 Test Requested and Test Method

測試項目 Test Items	參考方法 Reference Method
1. 性狀 Appearance	中國藥典, 2015 年版、第四部、通則 0104<br>The Pharmacopoeia of the People's Republic of China 2015, Vol.4, General chapter 0104
2. 水分 Determination of Water	中國藥典, 2015 年版、第四部、通則 0832<br>The Pharmacopoeia of the People's Republic of China 2015, Vol.4, General chapter 0832
3. 粒度 Size of Granule	中國藥典, 2015 年版、第四部、通則 0982<br>The Pharmacopoeia of the People's Republic of China 2015, Vol.4, General chapter 0982
4. 溶化性<br>Determination of Dispersibility	中國藥典, 2015 年版、第四部、通則 0104<br>The Pharmacopoeia of the People's Republic of China 2015, Vol.4, General chapter 0104
5. 裝量 Capacity	中國藥典, 2015 年版、第四部、通則 0942<br>The Pharmacopoeia of the People's Republic of China 2015, Vol.4, General chapter 0942
6. 重金屬及有害元素<br>Heavy Metals and Toxic Elements	中國藥典, 2015 年版、第四部、通則 2321；電感耦合等離子體質譜法；<br>The Pharmacopoeia of the People's Republic of China 2015, Vol.4, General chapter 2321；ICP-MS
7. 農藥殘留 Pesticides Residues	香港中醫藥管理委員會《中成藥註冊申請手冊》<br>Chinese Medicine Council of Hong Kong《Application Form: Registration of proprietary Chinese medicines》
8. 微生物限度 Microbial Limit	中國藥典, 2015 年版、第四部、通則 1105、1106、1107<br>The Pharmacopoeia of the People's Republic of China 2015, Vol.4, General chapter 1105，1106，1107
- 需氧菌總數 TAMC (Total Aerobic Microbial Count)	
- 霉菌和酵母菌總數 TYMC (Total Yeast and Mold Microbial Count)	
- 大腸埃希菌 Escherichia Coli	

****

編制 Report compiled by：[signature]　　審核 Reviewed by：[signature]

培力（南寧）藥業有限公司檢測中心 PuraPharm (Nanning) Pharmaceuticals Co.,Ltd. Testing Laboratory
郵編(Postal No.)：530007　電話(Tel)：0771-3218026　傳真(Fax)：0771-3216602

分析結果 Test Results

測試項目 Test Items	品質標準 Quality Specification	肉蓯蓉配方顆粒 Rou Cong Rong
1. 性狀 Appearance	本品為淺灰色至灰棕色顆粒；氣特異，味甜、微苦。The product is light grey to greyish brown granule, has characteristic odour, sweet and slightly bitter taste.	符合規定 Conform
2. 水分 Determination of Water	≤6.5% (W/W)	3.4%<br>符合規定 Conform
3. 粒度 Size of Granule	不能通過一號篩和能通過五號篩的顆粒和粉末總和不得超過 15% Sum of weight of granules that cannot pass through sieve No.1 and weight of powder that can pass through sieve No.5≤15%	8%<br>符合規定 Conform
4. 溶化性<br>Determination of Dispersibility	應符合規定 Shall meet the requirement	符合規定 Conform
5. 裝量 Capacity	應符合規定 Shall meet the requirement	符合規定 Conform
6. 重金屬及有害元素#<br>Heavy Metals and Toxic Elements		
- 銅 Copper (Cu)	不得過 150.00mg/kg<br>Cu≤150.00 mg/kg	0.693mg/kg<br>符合規定 Conform
- 砷 Arsenic (As)	不得過 41.67mg/kg 或 1500μg/日<br>As≤41.67mg/kg or 1,500μg/day	0.062mg/kg（2.23μg/日 day）<br>符合規定 Conform
- 鎘 Cadmium (Cd)	不得過 97.22mg/kg 或 3500μg/劑<br>Cd≤97.22mg/kg or 3,500μg/dose	0.004mg/kg（0.14μg/劑 dose）<br>符合規定 Conform
- 鉛 Lead (Pb)	不得過 4.97mg/kg 或 179μg/日<br>Pb≤4.97mg/kg or 179μg/day	0.070mg/kg（2.52μg/日 day）<br>符合規定 Conform
- 汞 Mercury (Hg)	不得過 1.00mg/kg 或 36μg/日<br>Hg≤1.00mg/kg or 36μg/day	<0.001mg/kg（<0.04μg/日 day）<br>符合規定 Conform
7. 農藥殘留#<br>Pesticides Residues		
- 艾氏劑及狄氏劑<br>Aldrin & Dieldrin	艾氏劑及狄氏劑兩者之和≤0.05 毫克/千克<br>Sum of aldrin & dieldrin≤0.05 mg/kg<br>（檢出限艾氏劑 1.77μg/kg,狄氏劑 2.43μg/kg<br>LOD aldrin 1.77μg/kg, dieldrin 2.43μg/kg）	未檢出 Not detected<br>符合規定 Conform
- 氯丹 Chlordane	順式-,反式及氧化氯丹之和≤0.05 毫克/千克<br>Sum of cis-,trans- & oxy-chlordane≤0.05 mg/kg<br>（檢出限順式氯丹 2.05μg/kg, 反式氯丹 1.81μg/kg, 氧化氯丹 2.21μg/kg<br>LOD cis- chlordane 2.05μg/kg, trans- chlordane 1.81μg/kg, oxy-chlordane 2.21μg/kg）	未檢出 Not detected<br>符合規定 Conform
- 滴滴涕 DDT	4,4'-滴滴涕, 2,4'-滴滴涕, 4,4'-滴滴伊及 4,4'-滴滴滴之和≤1.0 毫克/千克<br>Sum of *p, p*'-DDT, *o,p*'-DDT, *p,p*'-DDE, *p,p*'-TDE ≤1.0 mg/kg<br>（檢出限 4,4'-滴滴涕 3.73μg/kg, 2,4'-滴滴涕 4.48μg/kg, 4,4'-滴滴伊 2.29μg/kg, 4,4'-滴滴滴 2.88μg/kg<br>LOD *p, p*'-DDT 3.73μg/kg, *o,p*'-DDT 4.48μg/kg, *p,p*'-DDE 2.29μg/kg, *p,p*'-TDE 2.88μg/kg）	未檢出 Not detected<br>符合規定 Conform
- 異狄氏劑 Endrin	≤0.05 mg/kg<br>（檢出限 LOD 3.78μg/kg）	未檢出 Not detected<br>符合規定 Conform

****

編制 Report compiled by：[signature]　　審核 Reviewed by：[signature]

培力（南寧）藥業有限公司檢測中心 PuraPharm (Nanning) Pharmaceuticals Co.,Ltd. Testing Laboratory
郵編(Postal No.)：530007　電話(Tel)：0771-3218026　傳真(Fax)：0771-3216602

- 七氯 Heptachlor	七氯及環氧七氯之和≤0.05 毫克/千克<br>Sum of heptachlor & heptachlor epoxide≤0.05 mg/kg<br>（檢出限七氯 1.78μg/kg, 環氧七氯 1.97μg/kg<br>LOD heptachlor 1.78μg/kg, heptachlor epoxide 1.97μg/kg）	未檢出 Not detected<br>符合規定 Conform
- 六氯苯 Hexachlorobenzene	≤0.1 mg/kg<br>（檢出限 LOD 2.16μg/kg）	未檢出 Not detected<br>符合規定 Conform
- 六六六 Hexachlorocyclohexane	*α*-,*β*-及 δ-異構體之和≤0.3 毫克/千克<br>Sum of *α*-,*β*-,δ- isomers≤0.3 mg/kg<br>（檢出限 *α*-六六六 1.61μg/kg, *β*-六六六 2.96μg/kg, δ-六六六 1.43μg/kg<br>LOD *α*- HCH 1.61μg/kg, *β*- HCH 2.96μg/kg, δ- HCH 1.43μg/kg）	未檢出 Not detected<br>符合規定 Conform
- 林丹 Lindane	≤0.6 mg/kg<br>（檢出限 LOD 1.56μg/kg）	未檢出 Not detected<br>符合規定 Conform
- 五氯硝基苯 Quintozene	五氯硝基苯，五氯苯胺及甲基五氯硫基苯之和≤1.0 毫克/千克<br>Sum of quintozene, pentachloroaniline and methyl pentachlorophenyl sulphide≤1.0 mg/kg<br>（檢出限五氯硝基苯 1.92μg/kg, 五氯苯胺 1.59μg/kg, 甲基五氯硫基苯 1.33μg/kg<br>LOD quintozene 1.92μg/kg , pentachloroaniline 1.59μg/kg , methyl pentachlorophenyl sulphide 1.33μg/kg）	未檢出 Not detected<br>符合規定 Conform
8. 微生物限度#<br>Microbial Limit		
- 需氧菌總數 TAMC (Total Aerobic Microbial Count)	≤500cfu/g	<10cfu/g<br>符合規定 Conform
-霉菌和酵母菌總數 TYMC (Total Yeast and Mold Microbial Count)	≤100cfu/g	<10cfu/g<br>符合規定 Conform
- 大腸埃希菌 Escherichia Coli	不得檢出/克 Not detected/g	未檢出/克 Not detected/g<br>符合規定 Conform

****

註：Remarks

1、 報告無"檢驗/檢測報告專用章"和缺騎縫章無效。

Test report without the stamp of "For Test/Test Report Only" is not authorized.

2、 複製的報告未重新加蓋"檢驗/檢測報告專用章"及騎縫章無效。

Certified copy of test reportwithout the stamp of"For Test/Test Report Only" is not authorized.

3、 報告無編制、審核及本中心代表簽字無效。

Test report must be authorized by the signature of the bodies of preparation, verification and representative of the testing centre.

4、 報告塗改、缺頁無效。

Test report with modification or lacking of page(s) is not authorized.

5、 對報告若有異議，請於收到報告之日起十五日內向檢驗單位提出書面申訴，否則按認可檢驗報告處理。本中心異議受理電話 0771-3218026。

The client is aggrieved by the test result. A request for review shall state in writing the reasons relied upon and shall be made to the testing centre within 15 days after receipt of the authorized test report. No requirement will be accepted after the definite time. Inquiry hot line is 0771-3218026.

6、 送樣委託檢驗，樣品名稱為委託單位自報名稱，報告僅對來樣負責。部份複製檢驗/檢測報告無效。

Name of tested product is provided by the client. The report will refer only to the sample tested. Copy of partial of the test report is not authorized.

# - 其他微生物限度要求: Other requirements for Microbial Limit Test

1. 含動物類藥材（包括提取物）的中藥固體製劑，每 10g 不得檢出沙門菌。

TCM solid preparation containing animal raw materials (including extract): Absence of Salmonella (10g).

2. 含藥材原粉的中藥固體製劑，每 10g 不得檢出沙門菌；耐膽鹽革蘭陰性菌應小於 10^2cfu（1g）。

TCM solid preparation containing herbal raw powders: Absence of Salmonella (10g); Not more than 10^2cfu of Bile-tolerant gram-negative bacteria (1g).

*** End of Report 報告完 ***

培力（南寧）藥業有限公司檢測中心 **PuraPharm (Nanning) Pharmaceuticals Co.,Ltd. Testing Laboratory**
中國廣西南寧市高新技術開發區科園大道 46 號 No.46, Ke Yuan Road,Nanning New & High-tech Industrial Development Zone, Guangxi,China.

對"牛膝配方顆粒"之樣品之分析報告
Report on the submitted sample identified by the client - Niu Xi

樣品名稱 Product Description	牛膝配方顆粒 Niu Xi
樣品編號 Product Code	1063
樣品規格 Product Specification	200 克/瓶 g/ bottle
批號 Batch No.	A1501307
有效期至(年/月/日)Expiry Date（yyyy/mm/dd）	2018/11/22
本批數量 Quantity Produced	9094 瓶 bottles
樣品收到時狀態 Sample Receiving Condition	室溫下存放於密封塑膠樽原來包裝中<br>In sealed bottle of the original package under ambient condition.
製造商 Manufacturer	生產部 Production Department
委託檢驗單位 Inspected Entity	生產部 Production Department
來源地 Region of Origin	生產部 Production Department
目的地 Region of Destination	培力（南寧）藥業有限公司檢測中心<br>PuraPharm (Nanning) Pharmaceuticals Co.,Ltd. Testing Laboratory
檢驗日期(年/月/日)Testing Period（yyyy/mm/dd）	2015/11/30 - 2015/12/07

測試項目、分析方法及分析結果 Test Requested, Test Method and Test Results
請參考續頁。Please refer to the following page(s)

****

培力（南寧）藥業有限公司檢測中心代表簽名
Signed for and on behalf of PuraPharm (Nanning) Pharmaceuticals Co.,Ltd. Testing Laboratory

PURAPHARM (NANNING) PHARMACEUTICALS CO., LTD. TESTING LABORATORY 培力（南宁）药业有限公司检测中心 检测报告专用章 THE SPECIAL STAMP OF TEST REPORT 4501000241336

測試項目及分析方法 Test Requested and Test Method

測試項目 Test Items	参考方法 Reference Method
1. 性狀 Appearance	中國藥典, 2010 年版, 第一部, 附錄 I C<br>The Pharmacopoeia of the People's Republic of China 2010, Vol.1, Appendix I C
2. 水分 Determination of Water	中國藥典, 2010 年版, 第一部, 附錄Ⅸ H<br>The Pharmacopoeia of the People's Republic of China 2010, Vol.1, Appendix Ⅸ H
3. 粒度 Size of Granule	中國藥典, 2010 年版, 第一部, 附錄Ⅺ B<br>The Pharmacopoeia of the People's Republic of China 2010, Vol.1, Appendix Ⅺ B
4. 溶化性<br>Determination of Dispersibility	中國藥典, 2010 年版, 第一部, 附錄 I C<br>The Pharmacopoeia of the People's Republic of China 2010, Vol.1, Appendix I C
5. 裝量 Capacity	中國藥典, 2010 年版, 第一部, 附錄Ⅻ C<br>The Pharmacopoeia of the People's Republic of China 2010, Vol.1, AppendixⅫ C
6. 重金屬及有害元素<br>Heavy Metals and Toxic Elements	中國藥典, 2010 年版, 第一部, 附錄Ⅸ B；電感耦合等離子體質譜法；<br>The Pharmacopoeia of the People's Republic of China 2010, Vol.1, Appendix Ⅸ B；ICP-MS
7. 農藥殘留 Pesticides Residues	香港中醫藥管理委員會《中成藥註冊申請手冊》<br>Chinese Medicine Council of Hong Kong《Application Form: Registration of proprietary Chinese medicines》
8. 微生物限度 Microbial Limit	中國藥典, 2010 年版, 第一部, 附錄 XIII C<br>The Pharmacopoeia of the People's Republic of China 2010, Vol.1, Appendix XIII C
- 細菌數 Microbial Count	
- 霉菌及酵母菌<br>Mould and Yeast Count	
- 大腸埃希菌 Escherichia Coli	

****

編制 Report compiled by：[signature]　　審核 Reviewed by：[signature]

培力（南寧）藥業有限公司檢測中心 PuraPharm (Nanning) Pharmaceuticals Co.,Ltd. Testing Laboratory
郵編(Postal No.)：530007　電話(Tel)：0771-3218026　傳真(Fax)：0771-3216602

分析結果 Test Results

測試項目 Test Items	品質標準 Quality Specification	牛膝配方顆粒 Niu Xi
1. 性狀 Appearance	本品應為黃色至棕黃色顆粒，氣微，味微甜而稍苦澀。The product is yellow to yellowish brown granule, sligthly odour, slightly sweet taste and then slightly bitter and astringent taste.	符合規定 Conform
2. 水分 Determination of Water	≤6.0% (W/W)	4.6%<br>符合規定 Conform
3. 粒度 Size of Granule	不能通過一號篩和能通過五號篩的顆粒和粉末總和不得超過 15% Sum of weight of granules that cannot pass through sieve No.1 and weight of powder that can pass through sieve No.5≤15%	6%<br>符合規定 Conform
4. 溶化性<br>Determination of Dispersibility	應符合規定 Shall meet the requirement	符合規定 Conform
5. 裝量 Capacity	應符合規定 Shall meet the requirement	符合規定 Conform
6. 重金屬及有害元素#<br>Heavy Metals and Toxic Elements		
- 銅 Copper (Cu)	不得過 150.00mg/kg<br>Cu≤150.00 mg/kg	1.649mg/kg<br>符合規定 Conform
- 砷 Arsenic (As)	不得過 41.67mg/kg 或 1500μg/日<br>As≤41.67mg/kg or 1,500μg/day	0.044mg/kg（1.58μg/日 day）<br>符合規定 Conform
- 鎘 Cadmium (Cd)	不得過 97.22mg/kg 或 3500μg/劑<br>Cd≤97.22mg/kg or 3,500μg/dose	0.005mg/kg（0.18μg/劑 dose）<br>符合規定 Conform
- 鉛 Lead (Pb)	不得過 4.97mg/kg 或 179μg/日<br>Pb≤4.97mg/kg or 179μg/day	0.074mg/kg（2.66μg/日 day）<br>符合規定 Conform
- 汞 Mercury (Hg)	不得過 1.00mg/kg 或 36μg/日<br>Hg≤1.00mg/kg or 36μg/day	＜0.001mg/kg（＜0.04μg/日 day）<br>符合規定 Conform
7. 農藥殘留#<br>Pesticides Residues		
- 艾氏劑及狄氏劑<br>Aldrin & Dieldrin	艾氏劑及狄氏劑兩者之和≤0.05 毫克/千克<br>Sum of aldrin & dieldrin≤0.05 mg/kg<br>（檢出限艾氏劑 1.77μg/kg,狄氏劑 2.43μg/kg<br>LOD aldrin 1.77μg/kg, dieldrin 2.43μg/kg）	未檢出 Not detected<br>符合規定 Conform
- 氯丹 Chlordane	順式-,反式及氧化氯丹之和≤0.05 毫克/千克<br>Sum of cis-,trans- & oxy-chlordane≤0.05 mg/kg<br>（檢出限順式氯丹 2.05μg/kg, 反式氯丹 1.81μg/kg, 氧化氯丹 2.21μg/kg<br>LOD cis- chlordane 2.05μg/kg, trans- chlordane 1.81μg/kg, oxy-chlordane 2.21μg/kg）	未檢出 Not detected<br>符合規定 Conform
- 滴滴涕 DDT	4,4'-滴滴涕, 2,4'-滴滴涕, 4,4'-滴滴伊及 4,4'-滴滴滴之和≤1.0 毫克/千克<br>Sum of *p, p*'-DDT, *o,p*'-DDT, *p,p*'-DDE, *p,p*'-TDE ≤1.0 mg/kg<br>（檢出限 4,4'-滴滴涕 3.73μg/kg, 2,4'-滴滴涕 4.48μg/kg, 4,4'-滴滴伊 2.29μg/kg, 4,4'-滴滴滴 2.88μg/kg<br>LOD *p, p*'-DDT 3.73μg/kg, *o,p*'-DDT 4.48μg/kg, *p,p*'-DDE 2.29μg/kg, *p,p*'-TDE 2.88μg/kg）	未檢出 Not detected<br>符合規定 Conform
- 異狄氏劑 Endrin	≤0.05 mg/kg<br>（檢出限 LOD 3.78μg/kg）	未檢出 Not detected<br>符合規定 Conform

****

編制 Report compiled by：[signature] 審核 Reviewed by：[signature]

培力（南寧）藥業有限公司檢測中心 PuraPharm (Nanning) Pharmaceuticals Co.,Ltd. Testing Laboratory
郵編(Postal No.)：530007 電話(Tel)：0771-3218026 傳真(Fax)：0771-3216602

- 七氯 Heptachlor	七氯及環氧七氯之和≤0.05 毫克/千克<br>Sum of heptachlor & heptachlor epoxide≤0.05 mg/kg<br>（檢出限七氯 1.78μg/kg, 環氧七氯 1.97μg/kg<br>LOD heptachlor 1.78μg/kg, heptachlor epoxide 1.97μg/kg）	未檢出 Not detected<br>符合規定 Conform
- 六氯苯 Hexachlorobenzene	≤0.1 mg/kg<br>（檢出限 LOD 2.16μg/kg）	未檢出 Not detected<br>符合規定 Conform
- 六六六 Hexachlorocyclohexane	α-,β-及 δ-異構體之和≤0.3 毫克/千克<br>Sum of α-,β-,δ- isomers≤0.3 mg/kg<br>（檢出限 α-六六六 1.61μg/kg, β-六六六 2.96μg/kg, δ-六六六 1.43μg/kg<br>LOD α- HCH 1.61μg/kg, β- HCH 2.96μg/kg, δ- HCH 1.43μg/kg）	未檢出 Not detected<br>符合規定 Conform
- 林丹 Lindane	≤0.6 mg/kg<br>（檢出限 LOD 1.56μg/kg）	未檢出 Not detected<br>符合規定 Conform
- 五氯硝基苯 Quintozene	五氯硝基苯，五氯苯胺及甲基五氯硫基苯之和≤1.0 毫克/千克<br>Sum of quintozene, pentachloroaniline and methyl pentachlorophenyl sulphide≤1.0 mg/kg<br>（檢出限五氯硝基苯 1.92μg/kg, 五氯苯胺 1.59μg/kg, 甲基五氯硫基苯 1.33μg/kg<br>LOD quintozene 1.92μg/kg , pentachloroaniline 1.59μg/kg , methyl pentachlorophenyl sulphide 1.33μg/kg）	未檢出 Not detected<br>符合規定 Conform
8. 微生物限度#<br>Microbial Limit		
- 細菌數 Microbial Count	≤500cfu/g	20cfu/g<br>符合規定 Conform
- 霉菌及酵母菌<br>Mould and Yeast Count	≤100cfu/g	＜10cfu/g<br>符合規定 Conform
- 大腸埃希菌 Escherichia Coli	不得檢出/克 Not detected/g	未檢出/克 Not detected/g<br>符合規定 Conform

****

編制 Report compiled by： 審核 Reviewed by：

培力（南寧）藥業有限公司檢測中心 PuraPharm (Nanning) Pharmaceuticals Co.,Ltd. Testing Laboratory
郵編(Postal No.)：530007 電話(Tel)：0771-3218026 傳真(Fax)：0771-3216602

註：Remarks

1、 報告無"檢驗/檢測報告專用章"和缺騎縫章無效。

Test report without the stamp of "For Test/Test Report Only" is not authorized.

2、 複製的報告未重新加蓋"檢驗/檢測報告專用章"及騎縫章無效。

Certified copy of test reportwithout the stamp of"For Test/Test Report Only" is not authorized.

3、 報告無編制、審核及本中心代表簽字無效。

Test report must be authorized by the signature of the bodies of preparation, verification and representative of the testing centre.

4、 報告塗改、缺頁無效。

Test report with modification or lacking of page(s) is not authorized.

5、 對報告若有異議，請於收到報告之日起十五日內向檢驗單位提出書面申訴，否則按認可檢驗報告處理。本中心異議受理電話 0771-3218026。

The client is aggrieved by the test result. A request for review shall state in writing the reasons relied upon and shall be made to the testing centre within 15 days after receipt of the authorized test report. No requirement will be accepted after the definite time. Inquiry hot line is 0771-3218026.

6、 送樣委託檢驗，樣品名稱為委託單位自報名稱，報告僅對來樣負責。部份複製檢驗/檢測報告無效。

Name of tested product is provided by the client. The report will refer only to the sample tested. Copy of partial of the test report is not authorized.

# - 其他微生物限度要求: Other requirements for Microbial Limit Test

1.霉菌及酵母菌: 固體製劑及不含糖、王漿、蜂蜜的液體製劑或半固體製劑檢查霉菌；含糖、王漿、蜂蜜的液體製劑或半固體製劑檢查霉菌和酵母菌。

A)Mould and Yeast Count: For dose in solid form and liquid form not containing sugar, royal jelly, honey or semi-solid form, mould count should be tested. For dose in liquid form containing sugar, royal jelly, honey or semi-solid form, both mould count and yeast count should be tested.

2.含有動物類原藥材的口服製劑，不得檢出沙門氏菌；含動物角、王漿、蜂蜜、阿膠的口服製劑，則毋須進行有關檢定。

For oral preparations containing animal-origins raw ingredients, Salmonella should not be detected. For oral preparations containing animal horn, royal jelly, bee honey and CollaCoriiAsini, test for Salmonella may be exempted.

*** End of Report 報告完 ***

检驗報告 Test Report

編號 No.：（C）20161617　　日期 Date : 2016/10/10　　

培力（南寧）藥業有限公司檢測中心 **PuraPharm (Nanning) Pharmaceuticals Co.,Ltd. Testing Laboratory**

中國廣西南寧市高新技術開發區 科園大道 46 號

No.46, Ke Yuan Road,Nanning New & High-tech Industrial Development Zone, Guangxi,China.

對“麻子仁丸顆粒安慰劑”之樣品之分析報告

Report on the submitted sample identified by the client - Ma Zi Ren Wan Ke Li An Wei Ji

樣品名稱 Product Description	麻子仁丸顆粒安慰劑 Ma Zi Ren Wan Ke Li An Wei Ji
樣品編號 Product Code	3252
樣品規格 Product Specification	7.5g/袋 g/ bag
批號 Batch No.	A1601527
有效期至(年/月/日)Expiry Date（yyyy/mm/dd）	2019/09/28
本批數量 Quantity Produced	6840 袋 bags
樣品收到時狀態 Sample Receiving Condition	室溫下存放於密封鋁塑複合膜原來包裝中<br>In sealed lamination film package under ambient condition.
製造商 Manufacturer	生產部 Production Department
委託檢驗單位 Inspected Entity	生產部 Production Department
來源地 Region of Origin	生產部 Production Department
目的地 Region of Destination	培力（南寧）藥業有限公司檢測中心<br>PuraPharm (Nanning) Pharmaceuticals Co.,Ltd. Testing Laboratory
檢驗日期(年/月/日)Testing Period（yyyy/mm/dd）	2016/09/30 - 2016/10/10

測試項目、分析方法及分析結果 Test Requested, Test Method and Test Results

請參考續頁。Please refer to the following page(s)

****

培力（南寧）藥業有限公司檢測中心代表簽名

Signed for and on behalf of PuraPharm (Nanning) Pharmaceuticals Co.,Ltd. Testing Laboratory

培力（南寧）藥業有限公司檢測中心 PuraPharm (Nanning) Pharmaceuticals Co.,Ltd. Testing Laboratory

郵編(Postal No.)：530007　　電話(Tel)：0771-3218026　　傳真(Fax)：0771-3216602

測試項目及分析方法 Test Requested and Test Method

測試項目 Test Items	參考方法 Reference Method
1. 性狀 Appearance	中國藥典, 2015 年版, 第四部, 通則 0104<br>The Pharmacopoeia of the People's Republic of China 2015, Vol.4, General chapter 0104
2. 水分 Determination of Water	中國藥典, 2015 年版, 第四部, 通則 0832<br>The Pharmacopoeia of the People's Republic of China 2015, Vol.4, General chapter 0832
3. 粒度 Size of Granule	中國藥典, 2015 年版, 第四部, 通則 0982<br>The Pharmacopoeia of the People's Republic of China 2015, Vol.4, General chapter 0982
4. 溶化性<br>Determination of Dispersibility	中國藥典, 2015 年版, 第四部, 通則 0104<br>The Pharmacopoeia of the People's Republic of China 2015, Vol.4, General chapter 0104
5. 裝量差異 Variation in content	中國藥典, 2015 年版, 第四部, 通則 0104<br>The Pharmacopoeia of the People's Republic of China 2015, Vol.4, General chapter 0104
6. 重金屬及有害元素<br>Heavy Metals and Toxic Elements	中國藥典, 2015 年版, 第四部, 通則 2321；電感耦合等離子體質譜法；<br>The Pharmacopoeia of the People's Republic of China 2015, Vol.4, General chapter 2321；ICP-MS
7. 農藥殘留 Pesticides Residues	香港中醫藥管理委員會《中成藥註冊申請手冊》<br>Chinese Medicine Council of Hong Kong《Application Form: Registration of proprietary Chinese medicines》
8. 微生物限度 Microbial Limit	中國藥典, 2015 年版, 第四部, 通則 1105、1106、1107<br>The Pharmacopoeia of the People's Republic of China 2015, Vol.4, General chapter 1105，1106，1107
- 需氧菌總數 TAMC (Total Aerobic Microbial Count)	
- 霉菌和酵母菌總數 TYMC (Total Yeast and Mold Microbial Count)	
- 大腸埃希菌 Escherichia Coli	

****

編制 Report compiled by：[signature] 審核 Reviewed by：[signature]

培力（南寧）藥業有限公司檢測中心 PuraPharm (Nanning) Pharmaceuticals Co.,Ltd. Testing Laboratory
郵編(Postal No.)：530007 電話(Tel)：0771-3218026 傳真(Fax)：0771-3216602

分析結果 Test Results

測試項目 Test Items	品質標準 Quality Specification	麻子仁丸顆粒安慰劑 Ma Zi Ren Wan Ke Li An Wei Ji
1. 性狀 Appearance	顆粒劑應乾燥、顆粒均勻、色澤一致，無吸潮、軟化、結塊、潮解等現象。Granules should be dry, uniform in size and consistent in colour. There should be no moisture absorption, softening, clumping, deliquescence, etc.	符合規定 Conform
2. 水分 Determination of Water	≤6.5% (W/W)	3.0% 符合規定 Conform
3. 粒度 Size of Granule	不能通過一號篩和能通過五號篩的顆粒和粉末總和不得超過 15% Sum of weight of granules that cannot pass through sieve No.1 and weight of powder that can pass through sieve No.5≤15%	10% 符合規定 Conform
4. 溶化性 Determination of Dispersibility	應符合規定 Shall meet the requirement	符合規定 Conform
5. 裝量差異 Variation in content	應符合規定 Shall meet the requirement	符合規定 Conform
6. 重金屬及有害元素# Heavy Metals and Toxic Elements		
- 銅 Copper (Cu)	不得過 150.00mg/kg Cu≤150.00 mg/kg	1.824mg/kg 符合規定 Conform
- 砷 Arsenic (As)	不得過 41.67mg/kg 或 1500μg/日 As≤41.67mg/kg or 1,500μg/day	0.064mg/kg（2.30μg/日 day）符合規定 Conform
- 鎘 Cadmium (Cd)	不得過 97.22mg/kg 或 3500μg/劑 Cd≤97.22mg/kg or 3,500μg/dose	0.007mg/kg（0.25μg/劑 dose）符合規定 Conform
- 鉛 Lead (Pb)	不得過 4.97mg/kg 或 179μg/日 Pb≤4.97mg/kg or 179μg/day	0.099mg/kg（3.56μg/日 day）符合規定 Conform
- 汞 Mercury (Hg)	不得過 1.00mg/kg 或 36μg/日 Hg≤1.00mg/kg or 36μg/day	<0.001mg/kg（<0.04μg/日 day）符合規定 Conform
7. 農藥殘留# Pesticides Residues		
- 艾氏劑及狄氏劑 Aldrin & Dieldrin	艾氏劑及狄氏劑兩者之和≤0.05 毫克/千克 Sum of aldrin & dieldrin≤0.05 mg/kg （檢出限艾氏劑 1.77μg/kg,狄氏劑 2.43μg/kg LOD aldrin 1.77μg/kg, dieldrin 2.43μg/kg）	未檢出 Not detected 符合規定 Conform
- 氯丹 Chlordane	順式-,反式及氧化氯丹之和≤0.05 毫克/千克 Sum of cis-,trans- & oxy-chlordane≤0.05 mg/kg （檢出限順式氯丹 2.05μg/kg, 反式氯丹 1.81μg/kg, 氧化氯丹 2.21μg/kg LOD cis- chlordane 2.05μg/kg, trans- chlordane 1.81μg/kg, oxy-chlordane 2.21μg/kg）	未檢出 Not detected 符合規定 Conform
- 滴滴涕 DDT	4,4'-滴滴涕, 2,4'-滴滴涕, 4,4'-滴滴伊及 4,4'-滴滴滴之和≤1.0 毫克/千克 Sum of *p, p'*-DDT, *o,p'*-DDT, *p,p'*-DDE, *p,p'*-TDE ≤1.0 mg/kg （檢出限 4,4'-滴滴涕 3.73μg/kg, 2,4'-滴滴涕 4.48μg/kg, 4,4'-滴滴伊 2.29μg/kg, 4,4'-滴滴滴 2.88μg/kg LOD *p, p'*-DDT 3.73μg/kg, *o,p'*-DDT 4.48μg/kg, *p,p'*-DDE 2.29μg/kg, *p,p'*-TDE 2.88μg/kg）	未檢出 Not detected 符合規定 Conform
- 異狄氏劑 Endrin	≤0.05 mg/kg （檢出限 LOD 3.78μg/kg）	未檢出 Not detected 符合規定 Conform

****

編制 Report compiled by：[signature] 審核 Reviewed by：[signature]

培力（南寧）藥業有限公司檢測中心 PuraPharm (Nanning) Pharmaceuticals Co.,Ltd. Testing Laboratory
郵編(Postal No.)：530007 電話(Tel)：0771-3218026 傳真(Fax)：0771-3216602

- 七氯 Heptachlor	七氯及環氧七氯之和≤0.05 毫克/千克<br>Sum of heptachlor & heptachlor epoxide≤0.05 mg/kg<br>（檢出限七氯 1.78μg/kg, 環氧七氯 1.97μg/kg<br>LOD heptachlor 1.78μg/kg, heptachlor epoxide 1.97μg/kg）	未檢出 Not detected<br>符合規定 Conform
- 六氯苯 Hexachlorobenzene	≤0.1 mg/kg<br>（檢出限 LOD 2.16μg/kg）	未檢出 Not detected<br>符合規定 Conform
- 六六六 Hexachlorocyclohexane	α-,β-及 δ-異構體之和≤0.3 毫克/千克<br>Sum of α-,β-,δ- isomers≤0.3 mg/kg<br>（檢出限 α-六六六 1.61μg/kg, β-六六六 2.96μg/kg, δ-六六六 1.43μg/kg<br>LOD α- HCH 1.61μg/kg, β- HCH 2.96μg/kg, δ- HCH 1.43μg/kg）	未檢出 Not detected<br>符合規定 Conform
- 林丹 Lindane	≤0.6 mg/kg<br>（檢出限 LOD 1.56μg/kg）	未檢出 Not detected<br>符合規定 Conform
- 五氯硝基苯 Quintozene	五氯硝基苯，五氯苯胺及甲基五氯硫基苯之和≤1.0 毫克/千克<br>Sum of quintozene, pentachloroaniline and methyl pentachlorophenyl sulphide≤1.0 mg/kg<br>（檢出限五氯硝基苯 1.92μg/kg, 五氯苯胺 1.59μg/kg, 甲基五氯硫基苯 1.33μg/kg<br>LOD quintozene 1.92μg/kg , pentachloroaniline 1.59μg/kg , methyl pentachlorophenyl sulphide 1.33μg/kg）	未檢出 Not detected<br>符合規定 Conform
8. 微生物限度#<br>Microbial Limit		
- 需氧菌總數 TAMC (Total Aerobic Microbial Count)	≤500cfu/g	20cfu/g<br>符合規定 Conform
-霉菌和酵母菌總數 TYMC (Total Yeast and Mold Microbial Count)	≤100cfu/g	＜10cfu/g<br>符合規定 Conform
- 大腸埃希菌 Escherichia Coli	不得檢出/克 Not detected/g	未檢出/克 Not detected/g<br>符合規定 Conform

****

編制 Report compiled by：[signature]　　審核 Reviewed by：[signature]

培力（南寧）藥業有限公司檢測中心 PuraPharm (Nanning) Pharmaceuticals Co.,Ltd. Testing Laboratory

郵編(Postal No.)：530007　電話(Tel)：0771-3218026　傳真(Fax)：0771-3216602

註：Remarks

1、 報告無“檢驗/檢測報告專用章”和缺騎縫章無效。

Test report without the stamp of “For Test/Test Report Only” is not authorized.

2、 複製的報告未重新加蓋“檢驗/檢測報告專用章”及騎縫章無效。

Certified copy of test reportwithout the stamp of“For Test/Test Report Only” is not authorized.

3、 報告無編制、審核及本中心代表簽字無效。

Test report must be authorized by the signature of the bodies of preparation, verification and representative of the testing centre.

4、 報告塗改、缺頁無效。

Test report with modification or lacking of page(s) is not authorized.

5、 對報告若有異議，請於收到報告之日起十五日內向檢驗單位提出書面申訴，否則按認可檢驗報告處理。本中心異議受理電話 0771-3218026。

The client is aggrieved by the test result. A request for review shall state in writing the reasons relied upon and shall be made to the testing centre within 15 days after receipt of the authorized test report. No requirement will be accepted after the definite time. Inquiry hot line is 0771-3218026.

6、 送樣委託檢驗，樣品名稱為委託單位自報名稱，報告僅對來樣負責。部份複製檢驗/檢測報告無效。

Name of tested product is provided by the client. The report will refer only to the sample tested. Copy of partial of the test report is not authorized.

# - 其他微生物限度要求: Other requirements for Microbial Limit Test

1. 含動物類藥材（包括提取物）的中藥固體製劑，每 10g 不得檢出沙門菌。

TCM solid preparation containing animal raw materials (including extract): Absence of Salmonella (10g).

2. 含藥材原粉的中藥固體製劑，每 10g 不得檢出沙門菌；耐膽鹽革蘭陰性菌應小於 10^2cfu（1g）。

TCM solid preparation containing herbal raw powders: Absence of Salmonella (10g); Not more than 10^2cfu of Bile-tolerant gram-negative bacteria (1g).

*** End of Report 報告完 ***
